# Levels of Cu, Zn, and Se in Maternal and Cord Blood in Normal and Pathological Pregnancies: A Narrative Review

**DOI:** 10.3390/ijms27010161

**Published:** 2025-12-23

**Authors:** Radomir Aničić, Dejan Mihajlović, Jovana Kocić, Jovana Jagodić, Aleksandar Stojsavljević

**Affiliations:** 1 Clinic for Gynecology and Obstetrics “Narodni Front”, 11000 Belgrade, Serbia; radomir.anicic@gmail.com (R.A.); gasicjovana@gmail.com (J.K.); 2 Faculty of Medicine, University of Belgrade, 11000 Belgrade, Serbia; 3 Department for Gynecology and Obstetrics, Clinical Health Center Kosovska Mitrovica, Anri Dinana bb, 38220 Kosovska Mitrovica, Serbia; dejan.mihajlovic@med.pr.ac.rs; 4 Faculty of Chemistry, University of Belgrade, 11000 Belgrade, Serbia; 5 Innovative Centre of the Faculty of Chemistry, University of Belgrade, 11000 Belgrade, Serbia; aleksandars@chem.bg.ac.rs

**Keywords:** copper, zinc, selenium, pregnancy, umbilical cord, blood

## Abstract

Copper (Cu), zinc (Zn), and selenium (Se) play a pivotal role in pregnancy. Both a deficiency and an excess of Cu, Zn, and Se have deleterious consequences for the outcome of pregnancy. Accordingly, maintaining optimal levels of circulating Cu, Zn, and Se is critical for proper fetal growth and development. However, to our knowledge, this is the first narrative global review that not only summarizes Cu, Zn, and Se levels in maternal and cord blood but also examines their associations with multiple adverse pregnancy outcomes. Thus, this up-to-date review seeks to address these key questions. To achieve these goals, literature was collected from the past several decades from three relevant databases (PubMed, Scopus, and Cochrane Library), and rigorous exclusion and inclusion criteria were set for peer-reviewed studies that met the requirements for a final inclusion in the review analysis. In this study, data is presented on the levels of Cu, Zn, and Se in maternal and cord blood across the globe (herein used to suggest optimal maternal levels for Cu, Zn, and Se during a normal, healthy pregnancy), elemental differences between maternal and cord blood, and the fluctuations of their blood levels depending on the trimester of pregnancy. In addition, the review presents findings on the effects of Cu, Zn, and Se on birth weight and anthropometric parameters of newborns, as well as on preterm birth, preeclampsia, gestational diabetes mellitus, neural tube defects, and congenital heart defects.

## 1. Introduction

The total amount of trace elements makes up 0.01% (per dry weight) of the human body. Most trace elements are not essential for humans; in fact, even low levels of most trace elements have detrimental effects on human health [1]. Essential trace elements for humans are iron (Fe), copper (Cu), zinc (Zn), manganese (Mn), chromium (Cr^3+^), cobalt (Co), iodine (I), molybdenum (Mo), and selenium (Se) [2]. Essential trace elements are key nutrients that cannot be synthesized in the human body [3]. A lack of essential trace elements can lead to serious disruptions in the functioning of the body and even death [4]. On the other hand, an excess of an essential trace element also disrupts the body’s homeostasis and leads to numerous disorders, including cancer [5].

Pregnancy is a physiologically altered state in females characterized by an increased need for essential nutrients [6]. Copper, Zn, and Se are pivotal trace elements whose balance is necessary for normal pregnancy [7]. Consequently, disturbed levels of these trace elements could have a pathological outcome for a pregnancy [8]. According to the European Food Safety Agency (EFSA), adult women generally need 1.3 mg of Cu per day. During pregnancy, it is necessary to intake 1.5 mg of Cu per day [9]. The daily requirement for Zn is 8 mg/day for adult women. However, during pregnancy, a higher intake of Zn is recommended, 11 mg/day [10]. Adult women, including pregnant women, need 70 µg of Se per day [11]. According to the American College of Gynecologists and Obstetricians (ACOG), these amounts of Cu, Zn, and Se are usually met through a balanced diet. Still, in certain cases, supplementation can be necessary [12].

Numerous studies have examined high or low levels of essential trace elements in pregnancy. Thus, high blood levels of Cu are associated with low birth weight (LBW), preterm birth (PTB), and spontaneous abortions [13]. Low levels of blood Cu are associated with increased risks of miscarriage and PTB [14]. High blood levels of Zn can be lethal for the fetus, while low blood Zn levels are associated with preeclampsia (PE), LBW, PTB, etc. [15]. Selenium plays a dominant role in preventing oxidative stress, increasing the body’s antioxidant capacity in synergy with vitamin E and other antioxidants. This support effectively helps maintain the body’s homeostasis, particularly during pregnancy [16]. High blood Se levels are associated with an increased risk of congenital heart defects (CHDs). Low blood Se levels during pregnancy are associated with several complications, including gestational hypertension, gestational diabetes mellitus (GDM), preterm birth, miscarriage, and LBW [17]. However, some studies indicate that the available evidence is still inconclusive regarding the impact of these trace elements on the conditions mentioned above [18].

In this narrative review, the aim was to present data on the levels of Cu, Zn, and Se in maternal and cord blood worldwide, the elemental differences between maternal and cord blood, the fluctuations of their blood levels depending on the trimester of pregnancy, and differences in Cu, Zn, and Se levels between arterial and venous cord blood. An additional aim was to present key findings on the effects of Cu, Zn, and Se on birth weight and PTB, PE, GDM, neural tube defects (NTDs), and CHDs.

**Figure 1:**
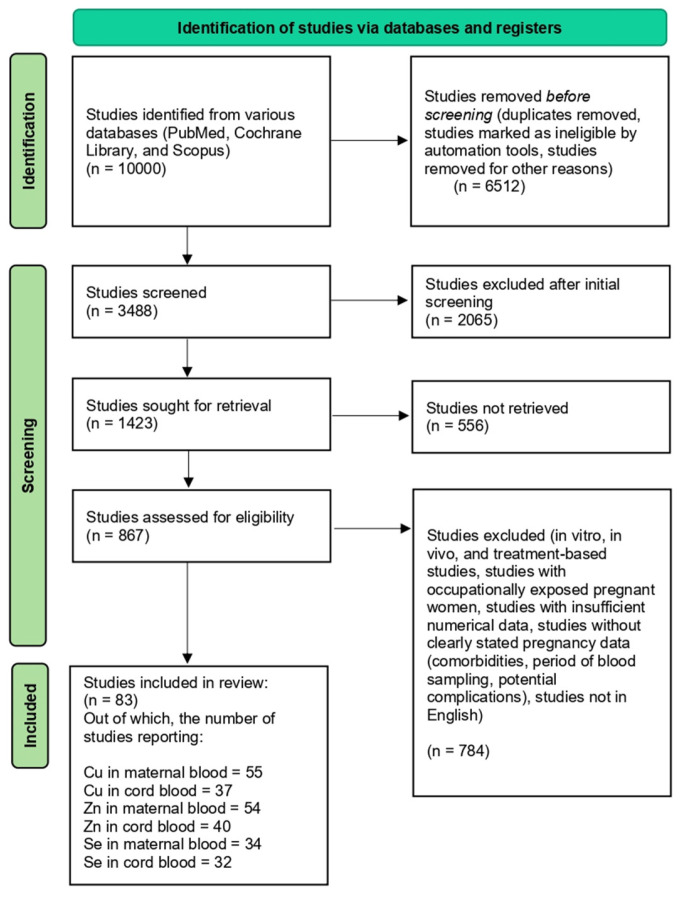
Study selection process for the review-flow diagram.

To resolve these goals, literature was collected from the past two and a half decades (from January 2000 to July 2025) from three relevant databases (PubMed, Scopus, and Cochrane Library), and rigorous exclusion/inclusion criteria were set for peer-reviewed studies that met the conditions for final inclusion in our review analysis. Studies were eligible for inclusion if they were peer-reviewed, reported original data, and assessed Cu, Zn, or Se levels in maternal and/or cord blood, with or without relevant clinical outcomes. Furthermore, they were assessed if they reported any of the following outcomes: birth weight, preterm birth, pre-eclampsia, gestational diabetes mellitus, neural tube defects, or congenital heart defects. Excluded articles encompassed letters, conference abstracts, commentaries, studies lacking trace element measurements, and publications without an accessible full text. All records were screened in two stages: title/abstract and full-text review, according to predefined eligibility criteria. Two reviewers independently screened studies, and discrepancies were resolved through discussion or consultation with a third reviewer. The overall study selection process, including the number of records identified, screened, excluded, and included, is documented in the PRISMA flow diagram (Figure 1). Search strategies were developed using combinations of controlled vocabulary (e.g., MeSH terms) and free-text keywords related to trace elements and pregnancy outcomes. The search terms included variations of: “copper,” “zinc,” “selenium,” “maternal blood,” “cord blood,” “pregnancy,” “trimester,” “birth outcomes,” “preterm birth,” “pre-eclampsia,” “gestational diabetes,” “neural tube defects,” and “congenital heart defects.” Boolean operators (AND/OR), truncation, and database-specific filters were applied to maximize the retrieval of relevant studies.

## 2. Copper, Zn, and Se Circulating Levels in Pregnant Women Around the World

This section included studies that examined Cu, Zn, and Se levels in maternal and cord blood from around the world. Some studies reported their numerical values as medians, while others opted for mean ± standard deviation. Copper, Zn, and Se levels in maternal and cord blood were considered only in healthy pregnant women, to: (1) conduct comparative analyses between countries and propose optimal ranges for Cu, Zn, and Se in whole blood and serum/plasma, (2) examine differences in Cu, Zn, and Se levels between maternal and cord blood, (3) propose optimal ranges for Cu, Zn, and Se depending on the trimester of pregnancy, and (4) examine differences in Cu, Zn, and Se levels between arterial and venous cord blood.

### 2.1. Maternal Blood Cu, Zn, and Se Levels Worldwide

**Figure 2:**
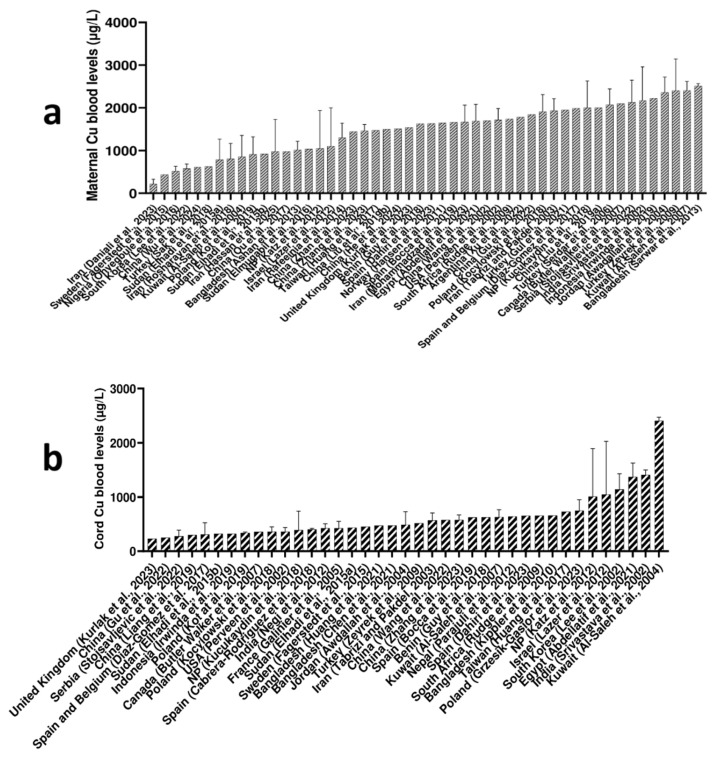
Reported global Cu levels (µg/L) in (**a**) maternal blood [19,20,21,22,23,24,25,26,27,28,29,30,31,32,33,34,35,36,37,38,39,40,41,42,43,44,45,46,47,48,49,50,51,52,53,54,55,56,57,58,59,60,61,62,63,64,65,66,67] and (**b**) cord blood [21,36,37,38,39,40,41,42,43,44,45,46,47,48,49,50,51,52,53,54,55,56,57,58,59,60,61,62,63,64,65,66,67,68,69,70,71,72,73]. Data are presented as mean and standard deviation (upper cup) or as median.

The observed global range of maternal Cu levels spans approximately from 400 to 2500 μg/L, with some of the highest levels reported in Bangladesh (2508 μg/L) [19], Kuwait (2404 μg/L) [20], and Turkey (2402 μg/L) [21]. The lowest maternal Cu levels were reported from Iran (213 μg/L) [22], Sweden (437 μg/L) [23], Nigeria (517 μg/L) [24], and South Korea (575 μg/L) [25], observing no apparent trend regarding parts of the world and reported low maternal Cu levels. Details are given in Figure 2a.

**Figure 3:**
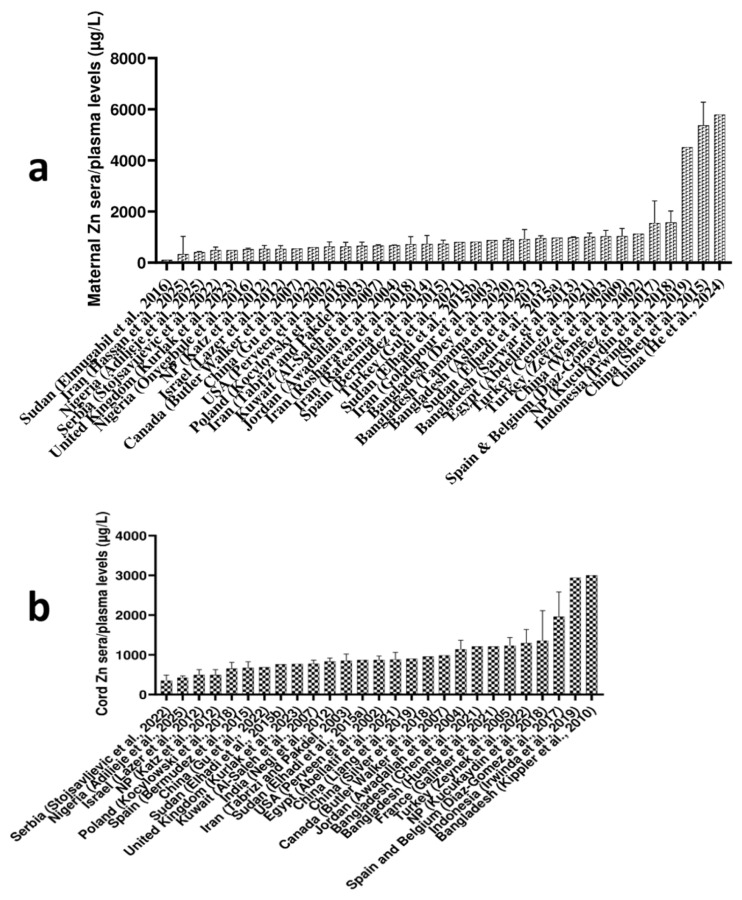
Reported global Zn levels (µg/L) in (**a**) maternal sera/plasma samples [21,26,27,28,29,30,31,32,33,34,35,36,37,38,39,40,41,42,43,44,45,46,47,48,49,50,51,52,53,54,55,56,57,58,59] and (**b**) cord sera/plasma samples [41,42,43,44,45,46,47,48,49,50,51,52,53,54,55,56,57,58,59,60,61,62,63,64,65,66,67]. Data are presented as mean and standard deviation (upper cup) or as median.

The highest levels of maternal Zn serum/plasma levels were observed in two studies from China (5368 μg/L, reported by Shen et al., 2015 and 5794 μg/L, reported by He et al., 2024) [26,27], followed by the Zn level reported in Indonesia (4516 μg/L) [28]. The data noted by these studies are markedly elevated, raising concerns about potential preanalytical errors that could have influenced the findings. He et al. (2024) reported that their participants resided in one of the world’s largest electronics manufacturing hubs [27], a factor that could account for the elevated Zn levels observed in their study. If the aforementioned studies from China and Indonesia are excluded, the majority of other studies worldwide fall into the loose range of 350–1500 μg/L for median Zn maternal sera/plasma levels. The lowest maternal sera/plasma Zn levels of 108 μg/L were shown in the study from Sudan [29], followed by 334 μg/L reported in Iran [30], and 416 μg/L reported in Nigeria [31]. Details are given in Figure 3a.

**Figure 4:**
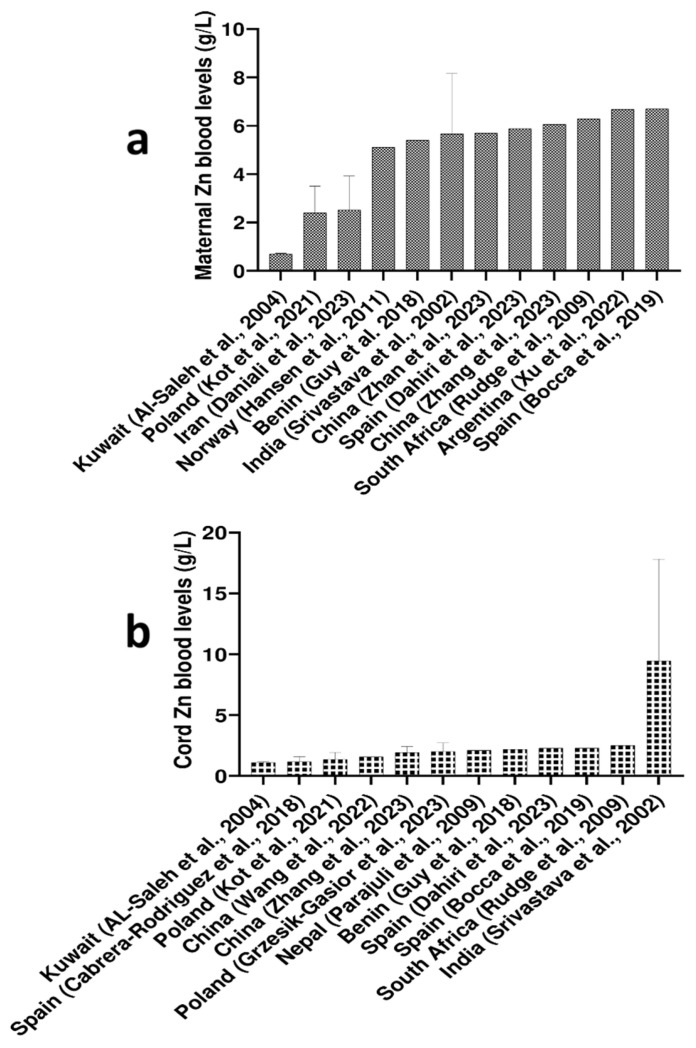
Reported global Zn levels (mg/L) in (**a**) maternal blood and [21,36,37,38,39,40,41,42,43,44,45,46] (**b**) cord blood [21,36,37,38,39,40,41,42,43,44,45,46]. Data are presented as mean and standard deviation (upper cup) or as median.

Since Zn levels differ between whole blood and serum/plasma, primarily due to Zn’s strong binding affinity to hemoglobin (Hb) within erythrocytes, which are absent in plasma or serum [32], we chose to report a separate global reference range for Zn concentrations in whole blood. Thus, considering maternal whole blood, the highest Zn levels were observed in Spain (6.70 mg/L) [33], followed by Argentina (6.68 mg/L) [34], and South Africa (6.29 mg/L) [35], while the lowest reported maternal blood Zn level came from Kuwait (0.696 mg/L) [36]. Details are given in Figure 4a.

**Figure 5:**
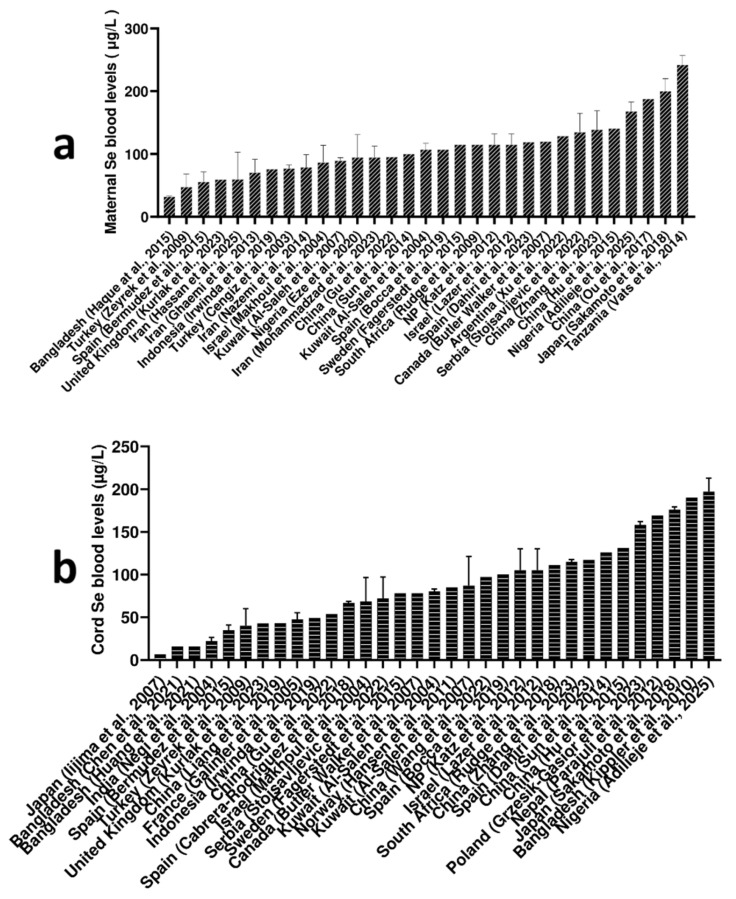
Reported global Se levels (µg/L) in (**a**) maternal blood [21,37,38,39,40,41,42,43,44,45,46,47,48,49,50,51,52,53,54,55,56,57,58,59,60,61,62,63,64,65] and (**b**) cord blood [31,45,46,47,48,49,50,51,52,53,54,55,56,57,58,59,60,61,62,63,64,65,66,67,68,69,70,71,72,73,74,75,76]. Data are presented as mean and standard deviation (upper cup) or as median.

Regarding maternal blood Se levels worldwide, Tanzania [37] showed the highest Se levels (242 μg/L), followed by Nigeria (168 μg/L) [31], China (141 μg/L) [38], and Serbia (135 μg/L) [39]. Globally, Bangladesh [40] showed the lowest Se maternal blood level with 32 μg/L, followed by Turkey (47.6 μg/L) [21]. Based on reported studies, maternal blood Se levels worldwide range from approximately 40 μg/L to 250 μg/L. Details are given in Figure 5a.

### 2.2. Cord Blood Cu, Zn, and Se Levels Worldwide

Some of the highest Cu cord levels were reported in Kuwait (2406 μg/L) [36], India (1410 μg/L) [41], and Egypt (1370 μg/L) [42]. Some of the lowest Cu cord levels were reported in the United Kingdom (232 μg/L) [43], China (250 μg/L) [44], and Serbia (276 μg/L). Based on the reported studies, Cu cord levels worldwide span approximately from 230 μg/L to 2400 μg/L [21,36,39,40,41,42,43]. Details are given in Figure 2b.

Globally, the highest Zn levels in cord sera/plasma (Figure 3b) were reported in Bangladesh (3000 μg/L) and Indonesia (2938 μg/L) [28,45]. In contrast, the lowest Zn cord sera/plasma levels were noted in Serbia (356 μg/L) and Nigeria (426 μg/L) [31,39]. Based on reported studies, excluding the two highest cord serum/plasma Zn levels from Asia, the global range of cord Zn levels generally spans from 350 μg/L to 1400 μg/L.

The highest levels of Zn in cord whole blood were observed in India (9.15 mg/L), while Zn levels in cord blood from other countries ranged from 1.10 to 2.50 mg/L (Figure 4b) [41]. Given the notably higher cord Zn levels (both in sera/plasma and whole blood) observed in South and South-East Asia, it can be hypothesized that these findings could be influenced by elevated Zn concentrations in local soil and groundwater, which could, in turn, affect Zn content in foodstuffs and ultimately contribute to the high Zn levels in cord blood [46].

Nigeria exhibited the highest global Se cord blood level (197 μg/L) among the reported studies, followed closely by Bangladesh (190 μg/L) [31,45]. According to the Nigerian study, the cord blood Se levels they observed were higher than the reference range, a finding they attributed to the efficient placental transfer of Se from mother to fetus [31]. Interestingly, two independent studies also reported the lowest global Se cord blood levels in Bangladesh, both noting the same value (15.8 μg/L) [47,48], followed by the Se level reported in India (22.1 μg/L) [49]. According to reported studies, if the lowest reported levels from two South Asian studies are excluded, the global range for cord blood Se broadly falls between 30 μg/L and 190 μg/L. Details are given in Figure 5b.

### 2.3. Differences in Levels of Cu, Zn, and Se Between Maternal and Cord Blood

Analysis of global data indicates that maternal blood Cu levels tend to be higher than those reported in cord blood (Figure 2 and Table 1). This can be explained by the strict regulation of Cu transfer from mother to fetus [50]. However, some exceptions exist. A study from Kuwait reported higher Cu levels in cord blood than in maternal blood (2406 μg/L vs. 853 μg/L, respectively) [36]. A study from Egypt observed higher maternal Cu levels overall but found relatively similar levels between maternal and cord blood (1630 μg/L vs. 1370 μg/L, respectively) [42].

Far more of the included studies assessed Zn levels in serum or plasma (36 studies reported maternal sera/plasma Zn levels, while 27 studies reported Zn levels in cord sera/plasma samples) than in whole blood (12 studies reported Zn levels in maternal whole blood samples, and 12 studies reported Zn levels in cord whole blood) (Figure 3 and Table 2). Zinc levels in both maternal and cord serum/plasma show considerable global variation. In several studies reporting relatively low maternal Zn levels, cord Zn levels remained relatively comparable, suggesting efficient fetal uptake [31,50,51]. The substantial inter-study variability worldwide could be attributed to factors such as differences in dietary habits and prenatal supplementation practices. Notably, the highest maternal Zn serum/plasma levels were reported in two independent studies from China, with levels exceeding 5000 μg/L, whereas the average levels worldwide generally range between 350 and 1500 μg/L [26,27]. Interestingly, most studies found higher Zn levels in cord serum/plasma than in corresponding maternal matrices [52,53,54]. Overall, the data suggest that Zn levels tend to be higher in cord serum/plasma than in maternal serum/plasma.

Considering Zn whole blood levels, results differed somewhat across countries. For instance, Al-Saleh et al. (2007 Kuwait) and Srivastava et al. (2002, India) reported higher Zn levels in cord blood than in maternal blood [20,41]. However, the majority of studies reported higher Zn levels in maternal whole blood than in cord whole blood, which was opposite to the trend observed in the sera/plasma samples (Figure 4 and Table 2). This likely reflects differences in how Zn is distributed between blood compartments: the placenta actively transports Zn to the fetus to support growth, particularly in late gestation, which increases extracellular (serum/plasma) Zn in cord blood, while intracellular Zn within fetal red blood cells remains comparatively lower.

The majority of studies reported that Se levels tend to be lower in cord blood than in maternal blood (Figure 4 and Table 3). This may be explained by the carefully regulated transfer of Se to the fetus, as well as the enhanced antioxidant defenses in the mother during pregnancy [50]. A study from Spain reported similar Se levels in maternal and cord blood (107 μg/L vs. 100 μg/L, respectively) [33]. However, some opposite trends were observed as well. For example, one study from China showed higher Se levels in cord blood (126 μg/L) than in maternal blood (100 μg/L), and a study from Nigeria exhibited the same trend (197 μg/L in cord blood vs. 168 μg/L in maternal blood) [31,55].

### 2.4. Copper, Zn, and Se Levels Depending on the Trimester of Pregnancy

Based on published data for Cu, Zn, and Se depending on the trimester of pregnancy (Table 1), Table 4 summarizes the levels of Cu, Zn, and Se in maternal blood depending on the trimester of pregnancy, while Table 5 suggests reference intervals for these trace elements in healthy, normal pregnancies. Our review of the literature revealed that Cu levels tend to increase as the pregnancy progresses. The lowest Cu level in the 1st trimester (1053 μg/L) was reported by Martin-Lagos et al., while a study by Polanska et al. (2017) reported the highest level (1980 μg/L) [56,57]. Overall, studies revealed a range of 1000 to 1900 μg/L Cu in maternal blood for the 1st trimester. In the 2nd trimester, Cu levels were in the range of 1100 to 2400 μg/L, while in the 3rd trimester, they were in the range of 1100 to 2600 μg/L (Table 4). The increase in Cu levels during pregnancy could be attributed to the expansion of maternal blood volume, the growing fetal demand for essential nutrients, and elevated ceruloplasmin concentrations driven by increased estrogen levels [58].

By reviewing the literature data in Table 2, it is notable that most studies reported a steady decrease in Zn levels as the pregnancy progresses, with two exceptions. A study by Choi et al. observed a decline in Zn levels from the 1st (66 μg/L) to the 2nd (55 μg/L) trimester, followed by an increase in the 3rd trimester (94 μg/L). In contrast, Huang et al. reported an opposite trend, with Zn levels rising from the 1st (1030 ± 270 μg/L) to the 2nd (1170 ± 450 μg/L) trimester, then decreasing in the 3rd trimester (1110 ± 280 μg/L) [70,71]. The general decrease in Zn levels from 1st to 3rd trimester could be explained by the increase in placental and fetal demand [72]. Taking into consideration the reported Zn levels in healthy, normal pregnancies, most studies fell into the loose ranges of 70 to 910 μg/L for the 1st, 70 to 810 μg/L for the 2nd, and 70 to 750 μg/L for the 3rd trimester (Table 2).

In contrast to Cu, our analysis revealed that Se levels tend to decrease as the pregnancy progresses (Table 3 and Table 4). These observations can likely be attributed to the utilization of Se from maternal blood to support fetal development and placental growth and function [72]. Based on the literature data, Se levels for the 1st trimester fell in the range of 45 to 110 μg/L, while levels in 2nd and 3rd trimesters ranged from 42 to 100 μg/L and 37 to 91 μg/L, respectively (Table 2).

### 2.5. Differences in Cu, Zn, and Se Levels Between Arterial and Venous Cord Blood

The umbilical cord usually contains two arteries and one vein. The umbilical arteries carry deoxygenated blood and metabolic waste from the fetus to the placenta, while the umbilical vein returns oxygenated, nutrient-rich blood from the placenta to the fetus. Together, they maintain essential fetal–placental circulation throughout gestation. Since arterial and venous blood do not have the same biochemical composition, the question arises whether the levels of Cu, Zn, and Se are the same or different [73,74]. In this regard, Al-Saleh et al. (2011) reported that trace element levels in cord venous serum were consistent with studies using mixed cord serum (arterial + venous) and with those studies using arterial and venous cord serum separately [36]. However, we found that the literature findings do not fully support their statement. Thus, Osada et al. (2002) found that Cu and Zn levels were significantly lower in umbilical artery serum (505 ± 149 µg/L and 406 ± 84.2 µg/L, respectively) than in umbilical vein serum (596 ± 71.8 µg/L and 465 ± 103 µg/L, respectively) in neonates of appropriate gestational age [75]. Their findings are consistent with the study by Rossipal et al. (2000) [76]. Interestingly, Díaz-Gómez et al. (2017) found lower levels of Zn in arterial cord serum (1548 ± 326 µg/L) than in venous cord serum (1959 ± 626 µg/L) but conversely found higher levels of Cu in arterial cord serum (451 ± 190 µg/L) than in venous cord serum (311 ± 216 µg/L) [54]. Al-Saleh et al. (2011) also demonstrated that Se levels in arterial cord serum were significantly higher than in venous cord serum (107 ± 7.20 vs. 80.4 ± 2.60 µg/L), while Rossipal et al. (2000) and Osada et al. (2002) did not find significant differences in Se levels in these matrices [36,75,76]. Lazer et al. (2012) investigated the differences in Cu, Zn, and Se levels in cord arterial and venous plasma during active labor and elective cesarean delivery [51]. In active labor, Cu levels were higher in the cord vein (1109 ± 885 μg/L) than in the cord artery (993 ± 812 μg/L). In contrast, during elective cesarean delivery, Cu levels were higher in the cord artery (905 ± 773 μg/L) compared to the cord vein (625 ± 658 μg/L). A similar pattern was observed for Se levels. In both active labor and elective cesarean delivery, Zn levels were consistently higher in cord artery plasma than in cord vein plasma [51]. Since few studies have addressed differences in Cu, Zn, and Se levels between arterial and venous cord serum/plasma, further research is urgently needed to provide more detailed insight into trace element levels in both serum and whole blood.

## 3. Copper, Zn, and Se Levels in Altered Pregnancy

In this section, the included studies consist of cross-sectional, case–control, retrospective, prospective cohort studies, and meta-analytical studies showing the influence of Cu, Zn, and Se on pregnancy outcomes, emphasizing fetal LBW and PTB, PE, GDM, NTDs, and CHDs.

### 3.1. Association Between Cu, Zn, and Se with Birth Weight and Neonatal Anthropometric Parameters

One indicator/predictor of fetal growth and development is birth weight. According to the World Health Organization (WHO), LBW has become one of the major risk factors for the global burden of disease [120]. The prevalence of LBW infants showed different geographic patterns, with rates of 7.6% in the United States, 5.9–11.8% in China, and about 25% in India [38]. The current rate of LBW stands at 8.2% of births in developed countries [121]. An estimated 20 million (15.5%) babies are born with LBW (<2.5 kg) worldwide each year, and approximately 95.5% of these births occur in low- and middle-income countries [114].

Birth weight primarily depends on genetics, placental circulation, and maternal nutrition [122]. Babies with LBW are about 20 times more likely to die during infancy and childhood than babies born with normal birth weight [114]. Intrauterine growth restriction and LBW are associated with increased morbidity and mortality, poor cognitive development, and neurological impairment, but also with widespread diseases later in life, including diabetes, cardiovascular diseases, renal dysfunction, mental disorders, and other complications [122].

Poor nutrition during pregnancy has been linked to miscarriages, stillbirths, and early neonatal mortality [123]. As premature babies are born before rapid intrauterine growth, they are susceptible to deficiencies of essential trace elements [124]. Micronutrient deficiencies are associated with pregnancy complications and anthropometric abnormalities in newborns [125]. In contrast, mineral supplementation in women with poor nutrition reduces the incidence of pregnancy complications [126].

Several studies investigated the relationship between Cu levels during pregnancy and birth outcomes, yielding inconsistent results (Table 1). Notably, Al-Saleh et al. (2011), Bermúdez et al. (2015), and Chen et al. (2021) reported a significant inverse relationship between cord blood Cu levels and birth weight, suggesting that elevated Cu levels could negatively affect fetal growth [36,47,77]. Bermúdez et al. (2015) further observed that cord Cu levels were significantly higher among small for gestational age (SGA) infants compared to appropriate for gestational age (AGA) and large for gestational age (LGA) groups [77]. Chen et al. (2021) mentioned that animal studies demonstrate growth restriction following high Cu exposure, such as in rat fetuses and fathead minnows, likely due to the downregulation of growth-related genes [47]. They also noted that elevated Cu exposure may pose a developmental risk [47].

In contrast, Awadallah et al. (2004) found no significant correlation between either maternal or cord Cu levels and birth weight [52]. Cabrera-Rodríguez et al. (2018) and Kippler et al. (2010) also found no significant association between cord Cu levels and birth weight, while Díaz-Gómez et al. (2017) found no significant correlation between maternal Cu levels and birth weight [45,54,78]. Overall, while some evidence points to a potential negative impact of elevated cord Cu levels on birth weight, findings remain inconclusive. Differences in study populations, dietary Cu exposure, matrices (plasma, serum, whole blood), and analytical methods could account for the variability in results. To establish a clearer understanding of how Cu influences fetal growth, future research should prioritize large-scale, prospective cohort studies with standardized sampling and measurement protocols.

Among the reviewed studies examining the relationship between Zn levels during pregnancy and birth weight, several reported a significant positive association (Table 2). Notably, Abdellatif et al. (2021) observed that higher Zn levels in cord blood were significantly correlated with increased birth weight [42]. Similarly, Awadallah et al. (2004) found a positive correlation between cord Zn levels and birth weight in healthy pregnancies [52]. These findings support the potential role of Zn in promoting fetal growth. Given Zn’s fundamental roles in antioxidant protection, immune competence, tissue formation, and cellular proliferation, all of which underpin normal fetal development, its potential impact on fetal growth and birth weight warrants close consideration.

In contrast, the majority of other studies, by Al-Saleh et al. (2011), Bermúdez et al. (2015), Bocca et al. (2019), Cabrera-Rodríguez et al. (2018), Chen et al. (2021), and Díaz-Gómez et al. (2017), found no significant link between Zn levels (in maternal or cord blood) and birth weight [33,36,47,54,77,78]. Additionally, Bocca et al. (2019) found no correlation between maternal and cord Zn levels, suggesting inconsistent transplacental transfer or homeostatic regulation [33]. While some studies—particularly those conducted in Middle Eastern and South Asian populations—indicate a positive association between Zn level and birth weight, the majority of findings from European cohorts do not support a consistent link. These inconsistencies could stem from regional variation in dietary Zn intake, underlying micronutrient profiles, socioeconomic factors, and environmental conditions. Differences in study methodologies, such as sample size, study design, timing, and type of Zn assessment, and how well confounders are accounted for, are also likely contributors. Taken together, these elements indicate that the association between Zn status and birth weight is highly context-specific and shaped by a complex mix of biological, environmental, and population characteristics.

**Figure 6:**
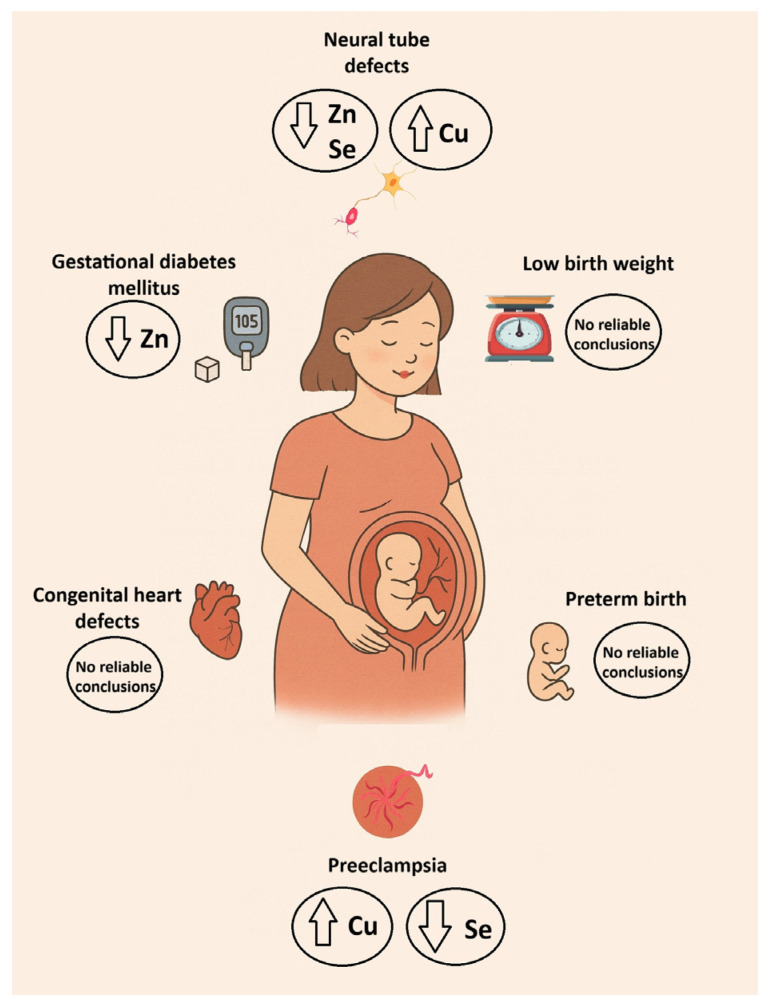
Pregnancy complications and their link with Cu, Zn, and Se.

Regarding Se, only Chen et al. (2021) reported a positive link between cord blood Se levels and newborn birth weight, suggesting that higher Se levels could contribute to increased birth weight (Table 3) [47]. In contrast, Al-Saleh et al. (2011), Bermúdez et al. (2015), Cabrera-Rodríguez et al. (2018), and Hu et al. (2015) found no significant relationship between maternal or cord blood Se levels and birth weight [36,38,77,78]. Al-Saleh et al. (2011) proposed that transplacental Se transfer occurs via passive diffusion and that monitoring Se levels in maternal or cord blood could not be useful for assessing fetal growth [36]. The majority of available evidence indicates that Se levels during pregnancy are not consistently linked to birth weight (Figure 6). Variation in findings across studies may reflect differences in geographic setting, underlying maternal nutritional status, and environmental sources of Se exposure. In addition, inconsistencies in the biological matrices used for Se assessment, laboratory analytical techniques, sample sizes, and overall study design could also contribute to the mixed results. Taking all of this into account, further large-scale studies are warranted to provide more comprehensive insights.

Several studies investigated the influence of Cu, Zn, and Se blood levels on neonatal anthropometric parameters, with mixed findings (Table 1, Table 2 and Table 3). Abdellatif et al. (2021) reported significant positive correlations between cord and maternal Cu levels with gestational age, birth length, and head circumference [42]. Similarly, Xu et al. (2022) found maternal Cu levels associated with shorter birth length and smaller head circumference [34]. Kot et al. (2021) observed a positive link between maternal Cu and gestational age, but a negative correlation with head circumference [127]. In contrast, Kippler et al. (2010) and Daniali et al. (2023) found no significant associations between Cu levels and gestational age or birth size [22,45]. Perveen et al. (2022) and Srivastava et al. (2002) also reported no significant differences in maternal Cu levels by gestational age, though Srivastava et al. (2002) noted a weak correlation between cord Cu and gestational age [41,88]. Grzesik-Gąsior et al. (2023) found the Cu/Zn ratio was significantly associated with head circumference [89]. Hassan et al. (2025) [30] reported negative correlations between serum Cu levels and anthropometric measures, especially head circumference. Details are given in Table 1.

Regarding Zn, Abdellatif et al. (2021) and Kippler et al. (2010) reported significant positive correlations between cord blood Zn levels and gestational age, birth length, and head circumference [42,45]. Abdellatif et al. also found that maternal Zn levels were positively associated with these parameters [42]. Similarly, Díaz-Gómez et al. (2017) and Kot et al. (2021) observed positive correlations between maternal Zn levels and gestational age, although Kot et al. (2021) noted a negative correlation between cord Zn and head circumference [54,127]. In contrast, Perveen et al. (2022) and Srivastava et al. (2002) [41] found no significant associations between plasma or cord Zn levels and gestational age [41,88]. Grzesik-Gąsior et al. (2023) and Daniali et al. (2023) also reported no significant associations between Zn levels and anthropometric outcomes [22,89]. Xu et al. (2022) found negative associations between maternal Zn levels and anthropometric parameters, although they observed a positive link with gestational age in female infants [34]. Details are given in Table 2.

Al Saleh et al. (2004) concluded that blood Se levels are not useful for assessing fetal growth. Similarly, Kippler et al. (2010) and Grzesik-Gąsior et al. (2023) reported no significant associations between Se levels and gestational age or anthropometric parameters [36,45,89]. In contrast, Makhoul et al. (2004) found a significant positive correlation between gestational age and cord serum Se levels [128]. Tang et al. (2016) and Xu et al. (2022) observed negative correlations between Se levels and anthropometric parameters [34,118]. Details are given in Table 3.

### 3.2. Association Between Cu, Zn, and Se and Preterm Birth

Preterm birth, defined as birth before 37 completed weeks of gestation, is the leading global cause of mortality in infants and children under 5 years of age [92]. Causes of PTB include decidual inflammation, decidual hemorrhage, pathologic uterine distention, and conditions that increase fetal and maternal stress [129]. According to two meta-analytic studies, trace elements could play a role in the pathogenesis of PTB [130,131]. Thus, Chiudzu et al. (2020) found that serum Cu levels were significantly higher in women who had spontaneous PTB (median, 2.61 mg/L) compared to women who delivered at term (median, 2.39 mg/L) [108]. In contrast, a study by Li et al. (2024) found no significant difference in serum Cu levels between cases (median, 1795 μg/L) and controls (median, 1823 μg/L), both of whom delivered at full term [107]. In the third study, conducted by Gohari et al. (2023), serum Cu levels were significantly lower in women who had preterm deliveries (1498 ± 531 μg/L) than in those who delivered at term (1840 ± 714 μg/L) [132]. Data related to Cu levels in cord serum reported by Galinier et al. (2005) showed that serum cord Cu levels were significantly lower in preterm than in term infants (full-term: 425 ± 133 µg/L vs. preterm: 286 ± 127 µg/L) [80]. On the other hand, Wang et al. (2022) reported the opposite results for cord blood Cu (cases: 540 μg/L vs. controls: 516 μg/L) [59]. Huang et al. (2021) in their study from Bangladesh showed that Cu levels in cord blood were not associated with the risk of PTB [48]. A multicountry meta-analysis conducted by Monangi et al. (2024) found that for every 1 μg/mL increase in maternal Cu level, the risk of PTB increased by 30% across 18 cohorts [133]. Although maternal Cu levels could be associated with PTB, Cu alone was not sufficient to predict or directly cause the condition. Copper is part of a broader interplay involving inflammation, oxidative stress, maternal nutrition, and genetic and environmental factors [134]. Low blood Cu levels can impair the body’s antioxidant defenses, compromise immune function, and increase susceptibility to infections. Copper is a cofactor for many enzymes, including superoxide dismutase, which protects cells from oxidative damage; low Cu levels can therefore lead to heightened oxidative stress, which could adversely affect placental function. Impaired immunity and increased infection risk can trigger inflammatory responses that compromise fetal membranes and the uterine environment. Together, these mechanisms have been linked to adverse pregnancy outcomes such as PTB and premature rupture of membranes, highlighting the possible role of adequate Cu levels in maintaining a healthy pregnancy [135]. Details are given in Table 1.

Regarding Zn, Chiudzu et al. (2020) found that serum Zn levels were higher in women who had spontaneous PTB (810 μg/L) than in those who delivered at term (730 μg/L) [108]. Li et al. (2024) did not find a statistically significant difference between serum Zn levels in cases (828 μg/L) and controls (813 μg/L) [107]. Huang et al. (2021) concluded that Zn levels in cord serum did not differ between preterm and full-term infants [48]. In contrast, Kucukaydin et al. (2018), in a study investigating the levels of trace elements in cord blood in cases of preterm premature rupture of membranes (PPROM), found that Zn levels were lower both in maternal blood and in the blood of preterm infants compared to levels in maternal blood without PPROM [72]. Specifically, maternal serum Zn levels were 800 ± 300 μg/L in the PPROM group vs. 1360 ± 740 μg/L in the non-PPROM group. In cord serum, Zn levels were 1170 ± 430 μg/L with PPROM compared to 1570 ± 450 μg/L without PPROM [72]. The overall results of the analyzed studies are contradictory and show differing data; therefore, a definitive conclusion about a significant link between Zn and PTB cannot be drawn. However, it remains possible that Zn, similarly to Cu, could play a role in the pathogenesis of PTB. Additional well-designed, large-scale studies are needed to clarify the potential contribution of Zn to PTB risk and to better understand the underlying biological mechanisms. Details are given in Table 2.

Chiudzu et al. (2020) reported that serum Se levels were higher, though not significantly, in women with spontaneous PTB (79.7 µg/L) compared with those who delivered at term (74.2 µg/L) [108]. Similarly, Li et al. (2024), in a study of 192 preterm and 282 full-term deliveries, found no significant difference in serum Se concentrations between the groups (preterm: 99.8 µg/L; term: 96.8 µg/L) [107]. Huang et al. (2021) also observed no significant difference in cord blood Se levels between preterm and term infants, consistent with their findings for other analyzed elements [48]. In contrast, a multicohort meta-analysis by Monangi et al. (2024), which included 17 cohorts comprising approximately 10,000 pregnant women, reported that although some studies identified a statistically significant correlation between higher maternal Se levels and reduced PTB risk, this association was not consistent across all populations [133]. The substantial heterogeneity observed suggests that contextual factors—such as nutritional status, sociodemographic characteristics, and genetic background—could influence the relationship between Se and PTB. Additional details are provided in Table 3.

### 3.3. Association Between Cu, Zn, and Se and Preeclampsia

Preeclampsia, as a subgroup of hypertensive disorders of pregnancy, is an entity associated with new-onset hypertension (>140/90 mm Hg systolic/diastolic blood pressure), usually with accompanying proteinuria (>300 mg/24 h) [118]. It commonly occurs after 20 weeks of gestation and often near term [136]. Preeclampsia encompasses 2% to 8% of pregnancy-related complications, more than 50,000 maternal deaths, and over 500,000 fetal deaths annually worldwide [137]. Nowadays, PE is considered as more than hypertension and proteinuria; it is now known to be a dysfunction of several organ systems (i.e., renal, hepatic, neurologic, hematological, or uteroplacental) caused by endothelial dysfunction [138]. Preeclampsia can be broadly categorized into two subtypes: early-onset (placental) and late-onset (maternal) PE [139]. Common risk factors, such as first pregnancy, multiple pregnancies, and pre-existing conditions such as hypertension and diabetes, can increase the risk of PE, as can maternal age (both younger and older than optimal), obesity, a family history of PE, and certain autoimmune conditions. In addition, assisted reproductive technologies, kidney disease, and specific infections are associated with a higher risk of PE [140]. Due to inadequate placentation and hypoxia, various factors with angiogenic and anti-angiogenic effects and oxidative stress parameters are elevated in PE [135]. To overcome this, specific trace elements are needed that would ultimately act synergistically with antioxidants and prevent further cell damage [18].

The majority of studies demonstrated significantly higher blood Cu levels in pregnant women with PE than in normotensive healthy pregnant women (controls) [34,49,96,97,98,99,100,101]. However, two studies reported contrasting findings, with lower serum Cu levels in PE pregnancies than in controls [19,141]. Regarding cord blood, Negi et al. (2012) found that Cu levels were significantly lower in cord plasma from women with PE (349 ± 95.3 µg/L) and eclampsia (261 ± 72.1 µg/L) than in controls (425 ± 82.3 µg/L) [49]. These results are consistent with a study from England, Kurlak et al., who reported higher serum Cu in women with PE (1606 μg/L) than in controls (1541 μg/L) [43]. Kurlak et al. [43] proposed that women who develop pre-eclampsia could exhibit increased uptake of essential trace elements such as Cu, potentially as a compensatory response to counteract the elevated oxidative stress associated with the condition. Thus, as Kurlak et al. [43] noted, higher Cu levels observed in such cases could be a consequence, rather than a cause, of pre-eclampsia [43]. On the other hand, no significant difference was found between early-onset and late-onset PE in maternal plasma Cu levels. In contrast, cord plasma Cu levels did differ between these two groups, with higher levels found in cases of late-onset PE compared to early-onset PE [43]. Details are given in Table 1.

Regarding Zn, most of the analyzed studies (n = 6) found no statistically significant difference in blood Zn levels between pregnant women with PE and controls [29,34,96,98,100,110]. Three studies reported higher Zn levels in PE women than in controls, while two studies observed lower Zn levels in the blood of PE women than in the blood of controls [24,27,99,141]. For example, Negi et al. (2012) showed that Zn levels were significantly lower in cord plasma from PE (744 ± 150 µg/L) and eclampsia (677 ± 117 µg/L) cases than in controls (830 ± 88.5 µg/L) [49]. Kurlak et al. [43] showed that maternal plasma Zn was significantly lower in 55 women with PE (418 µg/L) than in 60 controls (486 µg/L). Contrary to Cu, Kurlak et al. [43] found a significant difference in early-onset/late-onset PE, with lower blood Zn levels in both early-onset and late-onset PE compared with controls [43]. Zinc levels in cord plasma were significantly higher than maternal Zn levels in both PE groups, and there were no differences in circulating levels when the PE group was divided into early/late onset PE. Details are given in Table 2.

Regarding Se, all analyzed studies showed lower blood Se levels in women with PE than in controls [40,110,116]. Also, Negi et al. (2012) reported significantly lower cord blood Se levels in PE (18.6 ± 5.21 μg/L) and eclampsia (n = 14) (16.3 ± 5.23 μg/L) than in controls (22.2 ± 4.19 μg/L) [49]. These findings are consistent with those of Kurlak et al., who reported that Se levels in cord plasma were significantly lower (42.7 μg/L) than in maternal plasma (59.5 μg/L) [43]. Moreover, Se levels were also significantly lower in the cord plasma of newborns born to women with PE (37.4 µg/L) than in controls (42.7 µg/L). The increased oxidative stress observed in PE could contribute to reduced Se levels. In addition, PE causes impaired placental function, which could hinder the transfer of Se to the maternal circulation, further contributing to lower Se concentrations [43]. In contrast, Makhoul et al. (2004) in their cross-sectional study show that cord serum Se levels were significantly associated with birth weight and 5 min Apgar score, but not with PE [128]. Details are given in Table 3.

In summary, compared with healthy pregnancies, it can be concluded that Cu levels are significantly higher in the blood of pregnant women with PE, whereas Se levels are significantly lower (Figure 6). In the case of Zn, the findings are inconsistent, making it difficult to draw a definitive conclusion. Several factors could help explain this variability in Zn status. Inflammatory processes could influence the circulating levels of Zn in some cases. Differences in maternal nutrition, supplementation practices, and overall dietary intake across study populations can also influence Zn status. Additionally, oxidative stress could alter the activity of zinc-binding proteins, and the degree of placental dysfunction or disease severity could affect how Zn is handled during pregnancy [50]. Variations in study design, such as when samples were collected or how Zn was measured, likely contribute further to the mixed findings reported in the literature. These observations highlight the complex interplay of trace elements in PE and underscore the need for further well-designed, large-scale studies to better understand how imbalances in Cu, Se, and Zn could contribute to the pathophysiology of the disease and influence maternal and fetal outcomes.

### 3.4. Association Between Cu, Zn, and Se and Gestational Diabetes Mellitus

Gestational diabetes mellitus is a condition characterized by hyperglycemia during pregnancy in women who have not been previously diagnosed with diabetes [142]. The most common complications of GDM include macrosomia, which is associated with an increased risk of operative delivery and adverse neonatal outcomes, such as shoulder dystocia and its related complications [143]. Additionally, PE occurs more frequently in women with GDM. According to ACOG, all pregnant women should undergo laboratory-based screening for GDM, typically through blood glucose testing between 24 and 28 weeks of gestation (ACOG Practice Bulletin No. 190). However, for overweight or obese women who present additional risk factors—such as a previous history of GDM—early screening for undiagnosed type 2 diabetes is recommended, ideally at the first prenatal visit [144]. Many healthcare providers use a two-step approach for GDM screening, beginning with a 50 g oral glucose tolerance test (OGTT). Although the American Diabetes Association (ADA) acknowledges that Hb A1C testing could also be utilized, it is generally not recommended as a standalone diagnostic tool due to its lower sensitivity compared to OGTT [145].

There is evidence that trace elements could play a role in the pathogenesis of GDM [105]. We analyzed three studies concerning Cu levels in maternal blood, and all three showed different results. In one study, Cu levels in pregnant women with GDM were higher; in another, they were lower; and in the third, there was no difference compared to healthy pregnant women [106,119,146]. Details are given in Table 1.

Regarding Zn, the situation is somewhat different—most of the studies reviewed reported lower blood Zn levels in women with GDM compared to controls. Only one study, conducted by Mohammadzadeh et al. (2023), found no difference in serum Zn levels between the GDM group (69.20 μg/L) and the controls (67.57 μg/L) [104]. Details are given in Table 2.

Tan et al. (2001) analyzed serum Se levels in healthy pregnant women, those with impaired glucose tolerance, and those with GDM. They found that serum Se levels were significantly lower in women with GDM (63.5 ± 12 μg/L) than in controls (74.1 ± 16.7 μg/L) [119]. In contrast, Mohammadzadeh et al. (2023) reported different findings; they did not find a statistically significant difference in serum Se levels between women with GDM (98.2 ± 22.8 μg/L) and controls (94.7 ± 17.8 μg/L) [104]. Details are given in Table 3.

In summary, it can be concluded that the findings related to Cu and Se are inconsistent, and that further studies with larger sample sizes are needed to draw definitive conclusions about their association with GDM. As for Zn, there appears to be a pattern suggesting that maintaining optimal levels could be important in preventing its decline in GDM [147,148] (Figure 6). Zinc is essential for insulin synthesis, storage, and secretion, as well as for glucose metabolism and antioxidant defense, all of which are critical for maintaining glycemic control during pregnancy [104,149]. Therefore, ensuring optimal Zn status could help prevent or mitigate the decline in Zn levels often observed in women with GDM, potentially supporting better maternal and fetal outcomes.

### 3.5. Association Between Cu, Zn, and Se and Neural Tube Defects

Neural tube defects are birth defects of the brain and spinal cord that occur when the neural tube, which forms the brain and spinal cord, does not close completely during early pregnancy [146]. The defects arise from failure of embryonic neural tube closure by the fourth week of pregnancy [150]. NTDs occur in 0.5 to 2 per 1000 pregnancies [150]. The most common risk factor is a previous pregnancy with NTDs [146]. Periconceptional folate intake (4 mg/day of folic acid) can prevent about 70% of NTDs [151].

The summarized data of the literature search are given in Table 1, Table 2 and Table 3. Copper levels in the blood of women with NTD fetuses were significantly higher than those in healthy pregnant controls, which indicates that high levels of Cu could be associated with NTD development. Zeyrek et al. (2009) reported that Cu levels in both maternal and cord serum were significantly higher in the NTD group than in healthy controls [21]. Specifically, maternal serum Cu levels were 2831 ± 1017 μg/L in the NTD group vs. 2402 ± 744.2 μg/L in controls, while cord serum Cu levels were 790 μg/L in the NTD group vs. 517 μg/L in controls. In contrast, maternal serum Zn levels were significantly lower in the NTD group (836 ± 334 μg/L) than in controls (1036 ± 300 μg/L). However, cord serum Zn levels did not differ significantly between groups (NTD: 1391 ± 504 μg/L; controls: 1294 ± 346 μg/L). The Cu/Zn ratio in women’s sera of neonates with NTD was significantly higher than in controls, so this ratio could have a diagnostic value. The same authors showed no significant differences for Se levels in maternal serum (cases: 46.8 ± 26.4 μg/L vs. controls: 47.6 ± 20.6 μg/L) and cord serum (cases: 42.2 ± 21.9 μg/L vs. controls: 39.9 ± 20.0 μg/L) between NTD cases and controls [21]. In the study by Kucukaydin et al. (2018), who investigated Zn, Se, and Cu plasma levels in neonates diagnosed with NTDs and their mothers, plasma levels of Zn and Se were significantly higher in women from the control group compared to those from the NTD group [72]. Specifically, Zn levels were 1392 ± 111 μg/L in controls vs. 1084 ± 114 μg/L in the NTD group, and Se levels were 79.9 ± 8 μg/L in controls vs. 55.4 ± 7 μg/L in the NTD group. In contrast, Cu levels were significantly higher in the NTD group (2253 ± 245 μg/L) than in controls (1194 ± 295 μg/L). The same pattern was observed in cord plasma: Zn and Se levels were significantly lower in the NTD group than in controls, while Cu levels were significantly higher. Similar findings were reported by Cengiz et al. (2004), who showed that Cu serum levels were significantly higher in pregnant women whose babies had NTDs, compared to those with healthy babies (2331 ± 221 µg/L vs. 2073 ± 377 µg/L, respectively) [151]. In contrast, Zn serum levels were significantly lower in the NTD group (625 ± 159 µg/L) than in controls (1026 ± 237 µg/L) [151]. Golalipour et al. (2009) and Dey et al. (2010) reported the same trend regarding Zn; namely, Zn levels were significantly lower in pregnant women whose babies had NTDs compared to the control group [111,112]. Golalipour et al. (2009) reported maternal Zn levels of 745 μg/L in the NTD group vs. 877 μg/L in the control group [111]. Similarly, Dey et al. (2010) found that serum Zn levels were significantly lower in women with neonates who had NTDs (610 ± 53.1 μg/L) compared to women in the control group (883 ± 65.0 μg/L) [112]. Tian et al. (2022) showed that low maternal Se levels were associated with an increased risk of fetal malformations, including NTDs, and can negatively impact infant cognitive development [152]. Vats et al. (2014) and Cengiz et al. (2004) showed that blood Se levels measured in women with NTD neonates were significantly lower than in healthy pregnant women [37,151]. Specifically, Vats et al. (2014) reported the maternal blood Se level in NTD cases to be 306 ± 11.0 µg/L, while in controls it was 364 ± 17.1 µg/L [37]. Cengiz et al. (2004) reported the maternal serum Se level in NTD cases was 55.2 ± 11.3 µg/L, while in controls it was 77.4 ± 5.50 µg/L [151].

In summary, based on those aforementioned studies with consistent results, it can be concluded that pregnant women carrying a fetus with NTDs, as well as neonates with the same condition, exhibit higher blood levels of Cu and lower blood Zn and Se levels than do controls (Figure 6). The observed imbalances likely reflect the crucial roles of these trace elements in antioxidant defense, cell proliferation, and neural development during early fetal growth. These findings could potentially be of diagnostic significance in the future. Also, the diagnostic significance of the Cu/Zn ratio should be examined in the future.

### 3.6. Association Between Cu, Zn, and Se and Congenital Heart Defects

Congenital heart defects are the most common congenital anomalies in newborn babies (Xie et al. 2018) [102]. Screening for CHDs is a key component of the 2nd-trimester ultrasound, with >95% of pregnant women participating. The screening boasts high detection rates, identifying >80% of severe CHDs before birth [153]. Early detection enables accurate diagnosis and genetic testing, as well as timely planning and intervention [55].

Studies suggest that adequate intake of Zn and Se, and possibly maintaining an appropriate Zn/Cu or Se/Cu ratio, could be protective against CHDs. Yang et al. (2022) found that higher intakes of Zn and Se (from diet and supplements) during pregnancy could reduce the risk of CHDs, while no significant associations were found between dietary and supplement Cu intake with the risk of CHDs [154]. However, one study from China conducted by Guo et al. (2019) investigated the Se level in maternal hair and its link with CHDs [155]. They found that high levels of Se in maternal hair were associated with the presence of CHDs. Ou et al. (2017) measured Se levels in the blood of pregnant women whose babies had CHDs, and reported that the median blood Se level was significantly lower in the case group (pregnant women with babies diagnosed with CHDs) (172 μg/L) than in the control group (186 μg/L) [103]. Additionally, the same study demonstrated that the median maternal blood Cu level was significantly lower in the case group (838 μg/L) than in the control group (896 μg/L) [103] (Table 1, Table 2 and Table 3).

In summary, no reliable conclusions can be drawn about the benefits of Cu, Zn, and Se on CHDs, given the heterogeneity of the clinical matrices used by the authors and the small number of studies addressing this topic (Figure 6). Differences in population characteristics, nutritional status, environmental exposures, and the timing of trace element assessment could further contribute to the inconsistent findings. Given these limitations, additional well-designed, adequately powered studies are needed to clarify whether these essential trace elements play a meaningful role in the etiology or prevention of CHDs.

#### Limitations

Of the 83 studies analyzed, 34 controlled for key confounding factors such as dietary intake, supplementation, smoking status, BMI, parity, socioeconomic variables, genetic variants, and geographic differences. The limited and inconsistent adjustment for these variables represents an important limitation of the current evidence base. Nevertheless, the available data indicate that more comprehensive control of confounders would likely sharpen the estimates rather than substantially change the overall conclusions. In this sense, the main patterns observed across studies appear reasonably robust, though it remains essential to recognize that methodological constraints persist and should be taken into account when interpreting the findings. Differences in population characteristics, nutritional status, environmental exposures, and the timing of trace-element assessment may also contribute to the variability in findings. In light of these limitations, further well-designed and adequately powered studies are needed to better elucidate the role of trace elements in adverse pregnancy outcomes. Taken together, these constraints underscore the need for further large-scale, well-designed investigations that employ consistent and thorough adjustment for confounding factors.

## 4. Conclusions

To the best of our knowledge, this is the first study to examine the presented topic comprehensively. While Cu, Zn, and Se levels in blood-associated matrices during pregnancy differ across countries, they largely fall within a consistent range, enabling us to propose evidence-based global reference ranges for these essential trace elements in maternal blood and cord blood. Furthermore, most studies reported higher levels of Cu, Zn, and Se in maternal blood than in cord blood. Considering pregnancy trimesters, a clear trend can be observed regarding element levels: Zn and Se tend to decrease as the pregnancy progresses, while Cu levels increase. Only a few studies investigated differences in Cu, Zn, and Se levels between arterial and venous cord blood, and their findings are inconsistent. This highlights the urgent need for further research regarding this topic. Numerous studies have assessed the influence of Cu, Zn, and Se on neonatal anthropometry and birth outcomes, with mixed results. Evidence suggests potential associations between maternal trace element levels and preterm birth, although these are insufficient to establish predictive or causal relationships. More consistent patterns were observed for PE and GDM: women with PE generally exhibited higher Cu and lower Se levels, whereas women with gestational diabetes had lower Zn levels compared with healthy controls. Additionally, both pregnant women carrying fetuses with NTDs and affected neonates displayed higher Cu and lower Zn and Se levels than healthy controls. These findings raise important questions regarding the potential role of targeted trace element supplementation in the prevention and management of pregnancy complications.

Given these findings, further research is urgently needed. Large-scale, well-designed studies and meta-analyses are required to clarify the mechanistic roles of Cu, Zn, and Se in pregnancy and neonatal health. Future studies should prioritize standardized sampling and analytical protocols to improve comparability, employ longitudinal cohort designs to track trace element trajectories throughout pregnancy, and validate the proposed trimester-specific reference ranges in larger and more diverse populations. Additionally, investigations into environmental, dietary, and regional factors affecting trace element status are essential to understand the substantial variability observed across populations.

## Figures and Tables

**Table 1:** Levels of Cu, Zn, and Se in maternal blood depending on the trimester of pregnancy.

Ref.	Element	Biological Material	Analytical Technique	Pregnant Women		
				*N*	Trimester	Levels (µg/L)
[59]	Cu	Blood	AAS	-	1st	-
				1798	2nd	1168 ± 140
				2062	3rd	1176 ± 140
[60]	Cu	Plasma	AAS	36	1st	1893 ± 498
				49	2nd	2163 ± 475
				37	3rd	2145 ± 526
[61]	Cu	Serum	AAS	-	1st	1060 ± 120
				56	2nd	1070 ± 220
				-	3rd	1120 ± 145
[62]	Cu	Plasma	AAS	32	1st	1593.6 ^b^
				28	2nd	1830.4 ^b^
				18	3rd	1977.6 ^b^
[63]	Cu	Plasma	AAS	41	1st	1667 ± 418
				-	2nd	-
				35	3rd	2582 ± 566
[64]	Cu	Plasma	AAS	26	1st	866 ± 138
				23	2nd	1116 ± 279
				32	3rd	1140 ± 297
[57]	Cu	Plasma	AAS	392	1st	1980 ± 570
				307	2nd	2360 ± 590
				299	3rd	2550 ± 530
	Zn	Plasma	AAS	391	1st	910 ± 270
				304	2nd	780 ± 270
				299	3rd	740 ± 210
	Se	Plasma	AAS	410	1st	48.4 ± 10.5
				151	2nd	42.3 ± 9.08
				130	3rd	37.3 ± 9.79
[65]	Cu	Serum	AAS	73	1st	1475 ± 346
				30	2nd	1971 ± 240
				38	3rd	2042 ± 418
	Zn	Serum	AAS	73	1st	713 ± 129
				30	2nd	611 ± 86.3
				38	3rd	585 ± 115
	Se	Serum	AAS	73	1st	109 ± 20.1
				30	2nd	99.0 ± 24.4
				38	3rd	85.5 ± 12.8
[66]	Cu	Serum	HR-ICP-MS	291	1st	1575 (897–3660) ^a^
				555	2nd	1986 (1226–3435) ^a^
				491	3rd	2082 (1210–3750) ^a^
	Zn	Serum	HR-ICP-MS	291	1st	832 (577–1300) ^a^
				555	2nd	73.0 (53.4–103) ^a^
				491	3rd	71.0 (49.4–110) ^a^
	Se	Serum	HR-ICP-MS	291	1st	77.6 (44.0–112) ^a^
				555	2nd	76.0 (42.5–112) ^a^
				491	3rd	65.0 (38.0–105) ^a^
[67]	Cu	Serum	AAS	34	1st	1434 ± 971
				44	2nd	1650 ± 483
				52	3rd	1692 ± 755
	Se	Serum	AAS	34	1st	107 ± 15.8
				44	2nd	83.0 ± 17.4
				52	3rd	79.8 ± 19.0
[44]	Cu	Serum	ICP-MS	-	1st	1309 ± 435
				162	2nd	1720 ± 389
				-	3rd	1932 ± 285
	Zn	Serum	ICP-MS	-	1st	795 ± 150
				162	2nd	745 ± 161
				-	3rd	653 ± 149
[7]	Cu	Serum	AAS	177	1st	1323 ± 382
				174	2nd	1649 ± 400
				-	3rd	-
	Zn	Serum	AAS	177	1st	813 ± 319
				174	2nd	743 ± 225
				-	3rd	-
	Se	Serum	AAS	177	1st	44.9 ± 9.23
				174	2nd	47.2 ± 10.9
				-	3rd	-
[69]	Zn	Serum	AAS	60	1st	749 ± 91.0
				60	2nd	731 ± 106
				60	3rd	684 ± 99
[70]	Cu	Serum	AAS	32	1st	1340 ± 400
				40	2nd	1730 ± 330
				45	3rd	1210 ± 400
	Zn	Serum	AAS	31	1st	1030 ± 270
				44	2nd	1170 ± 450
				50	3rd	1110 ± 280
[56]	Cu	Serum	AAS	7	1st	1053 ± 498
				12	2nd	1616 ± 304
				19	3rd	1689 ± 344
	Zn	Serum	AAS	7	1st	829 ± 253
				12	2nd	846 ± 329
				19	3rd	620 ± 142
[71]	Cu	Serum	ICP-MS	52	1st	1120 ^b^
				97	2nd	1670 ^b^
				96	3rd	1780 ^b^
	Zn	Serum	ICP-MS	52	1st	66.0 ^b^
				97	2nd	55.0 ^b^
				96	3rd	94.0 ^b^
	Se	Serum	ICP-MS	52	1st	97.0 ^b^
				97	2nd	94.0 ^b^
				96	3rd	91.0 ^b^

^a^ Median (P2.5–P97.5); ^b^ Median. Abbreviations: AAS—atomic absorption spectrophotometry; ICP-MS—inductively coupled plasma mass spectrometry; HR—high resolution.

**Table 2:** Proposed optimal maternal blood levels for Cu, Zn, and Se during a normal, healthy pregnancy.

	1st Trimester	2nd Trimester	3rd Trimester
Cu (μg/L)	1000–1900	1100–2400	1100–2600
Zn (μg/L)	70–910	70–810	70–750
Se (μg/L)	45–110	42–100	37–91

**Table 3:** Copper (Cu) levels in maternal blood and umbilical cord blood of pregnant women worldwide (µg/L).

Reference	Study Design	Country	*N*	Biological Material	Analytical Technique	Cu Levels (µg/L)		Main Findings
						Maternal	Cord	
[42]	Cross-sectional	Cairo, Egypt	150 (40 preterm + 35 full-term) and their mothers	Serum	FAAS	Preterm: 1400 ± 330 Full-term: 1690 ± 390	Preterm: 750 ± 280 Full-term: 1370 ± 260	Maternal and cord serum Cu levels differed significantly and were positively correlated (ρ = 0.625). Cord Cu was higher in term than preterm neonates and differed between AGA and SGA infants. Cord Cu positively correlated with gestational age (ρ = 0.851), birth weight, length, and head circumference. Maternal Cu also correlated positively with gestational age and newborn anthropometrics, with higher maternal Cu linked to better outcomes
[36]	Cross-sectional	Kuwait	39	Blood	GFAAS	2406 ± 65.3	853 ± 54.5	Maternal Cu was higher than cord Cu (maternal/cord ratio 0.32 ± 0.03), with a negative correlation between cord Cu and birth weight. Transplacental Cu transfer appears to occur via passive diffusion. Maternal Cu/Zn ratio (3.60 ± 0.22 µg/L) exceeded cord levels (0.77 ± 0.09 µg/L).
[52]	Cross-sectional	Jordan	186	Serum	AAS	2360 ± 360	490 ± 240	Significantly higher levels of Cu in cord blood than in maternal blood. From the 1st to the 3rd trimester of pregnancy, serum Cu levels were significantly increased. No significant association between Cu levels in maternal and cord blood and birth weight (3.34 ± 0.44 kg).
[77]	Cross-sectional	Valencia, Spain	54	Plasma	ICP-MS	1460 ± 515	260 ± 85.1	A positive correlation between cord blood Cu levels and maternal blood (ρ = 0.484). Significant correlation between cord Cu levels and birth weight (r = −0.25). Cord blood Cu levels were significantly higher among SGA infants than in AGA and LGA infants.
[33]	Cross-sectional	Tarragona, Spain	53	Blood	SF-ICP-MS	1664	623	Significantly lower Cu levels in cord blood than in maternal blood, indicating limited transplacental transfer. Significantly higher Cu levels at delivery compared to the 1st trimester (1664 μg/L vs. 1302 μg/L). No significant correlation in Cu levels between maternal and cord blood.
[53]	Cross-sectional	Arctic Canada	352	Plasma	GF-AAS	2097	357	Maternal Cu levels were significantly higher than in cord blood.
[78]	Cross-sectional	Canary Islands, Spain	471	Blood	ICP-MS	-	402 ± 194	No significant relationship between cord Cu levels and birth weight.
[47]	Prospective	Bangladesh	745	Serum	ICP-MS	-	475	A significant negative association between cord blood Cu levels and birth weight.
[54]	Cross-sectional	Spain and Belgium	66	Serum	F-AAS	1988 ± 476	311 ± 216	Cord Cu was higher than maternal Cu, and Cu and Zn were negatively correlated (r = −0.35). Maternal Cu/Zn ratio exceeded cord levels, and maternal age correlated with the cord Cu/Zn ratio. Gestational age was positively associated with Cu, but Cu was not linked to birth weight.
[79]	Case–control	Sudan	104	Serum	F-AAS	Vaginal delivery: 788 Cesarean delivery: 924	Vaginal delivery: 435 Cesarean delivery: 322	Maternal Cu did not differ by delivery mode, but cord Cu was higher after vaginal delivery. Maternal and cord Cu levels were not significantly correlated.
[23]	Case–control	Sweden	80	Erythrocytes	ICP-MS	437	455	Cu levels did not differ between maternal and cord blood or between women with and without an anthroposophic lifestyle.
[80]	Cross-sectional	France	510 (262 full-term + 248 preterm)	Serum	FAAS	-	Full-term: 425 ± 133 Preterm: 286 ± 127	Cord blood Cu was lower in preterm than term infants, unaffected by gender, and increased steadily with gestational age from 26 to 42 weeks.
[81]	Cross-sectional	Benin	60	Blood	ICP-MS	1609	627	Cu levels were significantly lower in cord blood than in maternal blood. Blood levels of Cu were significantly higher in maternal blood (collected at delivery) than during the 1st trimester of pregnancy.
[82]	Cross-sectional	Taiwan	145	Blood	ICP-MS	1470	730	Maternal and cord Cu were positively correlated (ρ = 0.21), with cord Cu lower than maternal. Prenatal vitamin use (>3×/week) was linked to lower maternal Cu, and maternal age showed a borderline association with Cu levels.
[48]	Prospective	Bangladesh	745	Serum	ICP-MS	-	475	Cu levels in cord blood were not associated with the risk of preterm birth.
[28]	Cross-sectional	Jakarta, Indonesia	51	Serum	ICP-MS	Term: 2226 Preterm: 2153	Term: 322 Preterm: 206	Cu levels were higher in cord blood from the term group (n = 25) than from the preterm group (n = 26).
[83]	Case–control	NP	43 cases with severe PE and 80 healthy pregnant women	Serum	ICP-MS	Cases: 2239 ± 575 Controls: 1050 ± 877	Cases: 526 ± 211 Controls: 1109 ± 885	Maternal blood Cu levels were significantly higher in PE group than in controls. Cu levels were significantly lower in maternal and cord blood from the PE group than from controls
[45]	Cross-sectional	Bangladesh	44	Plasma	ICP-MS	-	660	Cord blood Cu levels were not significantly associated with birth weight, chest circumference, gestational age, birth length, and head circumference.
[84]	Cross-sectional	Greater Poland region	64	Serum	F-AAS	1910 ± 400	360 ± 90	Cord serum Cu levels were significantly lower than in maternal serum.
[85]	Cross-sectional	Gryfino, Poland	136	Blood	ICP-OES	910 ± 410	340 ± 130	Maternal Cu was over twice that of cord blood (*p* < 0.05), positively associated with gestational age, and negatively correlated with newborn head circumference (r = −0.29).
[72]	Prospective cohort	NP	68	Serum	FEAS	with PPROM: 2020 ± 670 without PPROM: 2010 ± 630	with PPROM: 340 ± 220 without PPROM: 390 ± 250	Cu levels did not differ between the PPROM (n = 35) and non-PPROM groups (n = 33) in either maternal serum or cord serum.
[51]	Prospective case–control	Israel	80	Plasma	ICP-MS	Active: 1050 ± 877 Elective CD: 1046 ± 835	Active: 1109 ± 885 Elective CD: 625 ± 658	Significantly higher Cu levels in cord blood during active labor compared to elective cesarean delivery (CD).
[25]	Cross-sectional	Gwangju, South Korea	81	Plasma	AAS	1140 ± 297	575 ± 109	Plasma Cu levels in the 1st, 2nd, and 3rd trimesters were 866 ± 138, 1116 ± 279 and 1140 ± 297 µg/L, respectively. Plasma Cu levels were significantly higher during the 2nd and 3rd trimesters than in the 1st. Cu levels in cord blood were about 50% lower than in maternal blood (*p* < 0.05).
[86]	Cross-sectional	Ma’anshan, China	3416	Serum	ICP-MS	the 1st trimester: 1530 the 2nd trimester: 2030	300	Cord serum Cu was lower than maternal serum, and Cu levels differed between trimesters. Multivitamin use during pregnancy was positively associated with maternal Cu.
[49]	Case–control	Varanasi, India	33 cases and 18 controls	Plasma	Spectrophotometry	-	425 ± 82.3	Copper levels were significantly lower in cord blood from women with PE (n = 19) (349 ± 95.3 µg/L) and eclampsia (n = 14) (261 ± 72.1 µg/L) than in controls (424 ± 82.3 µg/L).
[87]	Cross-sectional	Terai, Nepal	100	Blood	ICP-MS	-	643	Cord blood Cu levels were not associated with maternal age, socioeconomic status, living environment, or tobacco smoking.
[88]	Cross-sectional	Manhasset, New York, USA	35	Plasma	AAS	1715 ± 268	360 ± 77	No significant differences in maternal plasma Cu levels with gestational age. However, the maternal/cord plasma Cu ratio increased with gestational age.
[35]	Cross-sectional	South Africa	62	Blood	ICP-MS	1730	657	No significant association between maternal and cord blood Cu levels.
[26]	Prospective cohort	China	1568	Serum	AAS	1970 ± 383	-	Serum Cu levels increased significantly from the 1st to the 3rd trimester and were significantly higher than pre-pregnancy levels. Serum Cu levels were significantly lower in the group with PROM than in controls.
[89]	Cross-sectional	Lucknow, India	54	Blood	FAAS	2170 ± 790	1410 ± 900	Maternal Cu was higher and cord Cu slightly lower in low birth weight infants, but not significantly. Cu levels did not differ by gestational age, though cord Cu showed a weak positive correlation with gestational age.
[41]	Cross-sectional	Serbia	125	Plasma	ICP-MS	2127 ± 523	276 ± 114	Cu levels differed significantly between maternal and cord blood and were positively correlated. Cord Cu was higher in infants of mothers aged 20–25 than 31–35 years.
[90]	Longitudinal	Khoy, Iran	162	Serum	ICP-MS	1932 ± 285	570 ± 138	Differences in sera Cu levels during the 1st, 2nd, and 3rd trimesters (1309 ± 435, 1720 ± 389, 1932 ± 285 μg/L, respectively). Cu levels in cord blood serum were significantly lower than in maternal serum.
[21]	Case–control	Harran, Turkey	144	Serum	AAS	Cases: 2831 ± 1017 Controls: 2402 ± 744	Cases: 790 Controls: 517	Copper levels in maternal and cord serum in the NTD group were significantly higher than in controls. The Cu/Zn ratio in mothers of neonates with NTD was significantly higher (3.80 ± 2.10 vs. 2.60 ± 1.20) than in controls.
[91]	Cross-sectional	Seville, Spain	100	Blood	ICP-MS	1632	655	Cu levels in the women’s blood were almost three times higher than those found in cord blood (*p* < 0.05).
[92]	Cross-sectional	Lublin, Poland	134	Blood	HR-ICP-OES	-	750 ± 204	A positive association between cord blood Cu levels and weight gain in pregnant women. The Cu/Zn ratio was significantly associated with the head circumference of the newborn.
[91]	Prospective cohort	Beijing, China	48	Plasma	ICP-MS	1845	250	Cu levels increased during pregnancy (1273 vs. 1797 vs. 1845 µg/L). Cu levels were significantly lower in cord than in maternal plasma.
[93]	Prospective cohort	Norway	211	Blood	SF-ICP-MS	1650	-	Blood Cu levels increased significantly during pregnancy and decreased 6 weeks postpartum.
[93]	Longitudinal	Shanghai, China	100	Blood (at 4 time points: preconception, GW 16, 24, and 32)	ICP-MS	1464 ± 147	577 ± 93.0	Maternal blood Cu levels were higher during pregnancy than in the preconception period. Maternal blood Cu levels correlated between preconception and 24 weeks of gestation. From conception to the 3rd trimester, maternal Cu levels were positively correlated.
[43]	Case–control	Nottingham, England	55 cases with PE, 60 healthy normotensive pregnant women, and 30 healthy non-pregnant women	Plasma	ICP-MS	1541	232	Maternal plasma Cu was higher in women with PE (1606 µg/L) than in controls (1541 µg/L), with no difference between early- and late-onset PE. Cord Cu levels were lower than maternal levels but higher in infants of PE mothers (335 µg/L) than controls (232 µg/L). Elevated cord Cu was significant in late-onset PE only.
[30]	Case–control	Kermanshah province, Iran	57 cases with diabetes and 54 controls	Serum	ICP-MS	Cases: 1020 ± 710 Controls: 980 ± 746	-	No significant differences in maternal serum Cu between cases and controls. In controls, only head circumference showed a significant negative correlation with Cu (ρ = −0.33). In cases, Cu levels were negatively correlated with growth measures, particularly birth weight (ρ = −0.374) and head circumference (ρ = −0.345). Multivariate analysis showed that in women with diabetes, higher Cu levels adversely affected neonatal weight and head circumference.
[94]	Case–control	Fujian, China	120 cases with spontaneous preterm birth and 120 controls	Blood	ICP-MS	Cases: 650.87 Controls: 618.94	-	Blood Cu levels were significantly higher in spontaneous preterm birth cases than in controls. Note: Maternal blood was collected between 10 and 13 weeks of gestation.
[59]	Case–control	Guangdong, China	515 cases with preterm births (PTB) and 595 controls	Blood	ICP-MS	-	Cases: 540 Controls: 516	Cord blood Cu levels were significantly higher in cases than in controls. Cord blood Cu levels were positively associated with PTB; it was hypothesized that Cu could be a risk factor for PTB at relatively high levels.
[34]	Cross-sectional	Argentina	696	Blood (36 ± 12 h postpartum)	ICP-MS	1782	-	Blood Cu levels decreased with higher parity but increased with maternal age. Higher maternal Cu was linked to longer gestation, as well as lower birth weight, shorter neonatal length, and smaller head circumference.
[22]	Cross-sectional	Isfahan, Iran	263	Blood (collected during the 1st trimester)	ICP-MS	213.94 ± 115.85	-	No significant association between blood Cu levels and birth size outcomes.
[95]	Retrospective	Guangzhou, China	11,222 pregnant women and 545 non-pregnant women	Blood	ICP-MS	1447.7	-	Cu levels were higher in pregnant women than in controls, and at 12 weeks’ gestation, older pregnant women (>35) had higher Cu levels than younger women (<35)
[27]	Cross-sectional	Dongguan City, China	271 pregnant women, of whom 97 were diagnosed with mild PE, 64 with severe PE, and 110 were normotensive healthy pregnant women.	Serum	ICP-MS	1364	-	Serum Cu was lower in controls (1364 μg/L) than in women with PE, increasing from mild PE (1940 μg/L) to severe PE (2841 μg/L). These results suggest that higher Cu levels may contribute to the onset and severity of PE.
[96]	Cross sectional	Dhaka, Bangladesh	44 PE, 33 eclampsia, and 27 normotensive healthy pregnant women	Serum	AAS	1016 ± 127	-	Maternal serum Cu was highest in controls (1016 ± 127 μg/L), slightly lower in PE (1110 ± 127 μg/L), and lowest in eclampsia (952 ± 127 μg/L), with significant differences among groups.
[97]	Prospective	Diyarbakır, Turkey	179 pregnant women, 58 healthy pregnant women, 71 mild PE, 26 severe PE, and 24 HELLP (Hemolysis, Elevated Liver enzymes, and Low Platelet count, a form of severe preeclampsia) syndrome	Serum	AAS	626	-	Serum Cu was highest in HELLP (2099 ± 286 μg/L), followed by severe PE (1602 ± 208 μg/L), mild PE (812 ± 118 μg/L), and lowest in healthy pregnancies (626 ± 256 μg/L). All differences were significant, showing Cu levels rise with the severity of hypertensive disorders.
[29]	Case–control	Sudan	50 PE cases and 50 healthy pregnant women	Serum	AAS	1036	-	No significant differences in Cu levels between PE group (1116 μg/L) and controls (1036 μg/L).
[98]	Observational case–control	Istanbul, Turkey	88 pregnant women (in their 3rd trimester) included 43 preeclampsia patients and 45 normotensive pregnant women as controls.	Serum	Spectrophotometry	1952	-	Cu levels increased during pregnancy. PE cases had significantly higher Cu levels (2245 μg/L) than controls (1952 μg/L)
[99]	Case–control	Sanliurfa, Turkey	24 women with PE and 44 normotensive pregnant women	Plasma	AAS	31.60 ± 11.74 µg/g protein	-	Cu levels in PE group (47.9 ± 19.8 µg/g protein) were significantly higher than in controls (31.60 ± 11.74 µg/g protein).
[24]	Case–control	Nnewi, Nigeria	Of the total 102 pregnant women, 48 were non-preeclamptic while 54 were with PE	Serum	AAS	534 ± 114	-	Cu levels in PE group (1055 ± 201 μg/L) were significantly higher than in controls (534 ± 114 µg/L).
[100]	Case–control	Gorgan, Iran	100 pregnant women, 50 healthy pregnant women and 50 women with PE	Serum	AAS	1300 ± 340	-	Cu levels in PE women (2400 ± 640 μg/L) were significantly higher than in controls (1300 ± 340 μg/L).
[19]	Case–control	Bangladesh	50 cases of PE, with gestational period > 20 weeks and 58 normotensive pregnant women	Serum	AAS	2580 ± 60.0	-	Copper levels in PE women (1980 ± 100 μg/L) were significantly lower than in controls (2580 ± 60.0 μg/L).
[101]	Case–control	Bursa, Turkey	Thirty healthy, 30 mild PE and 30 severe PE pregnant women (31–38 week of pregnancy)	Serum	Spectrophotometry	1590 ± 380	-	Cu levels in the severe PE group (1940 ± 520 μg/L) were higher than in the mild PE group (1880 ± 480 μg/L). Both groups had significantly higher Cu levels than healthy controls (1590 ± 380 μg/L).
[102]	Case–control	Ankara, Turkey	14 pregnant women with babies with NTDs in 2nd trimester and 14 pregnant women with ultrasound- normal fetuses as a control group	Serum	AAS	2073 ± 377	-	Cu levels in cases (2331 ± 221 µg/L) were significantly higher than in controls (2073 ± 377 µg/L)
[103]	Case–control	China	112 pregnant women with babies with CHD and 109 pregnant women as a control	Plasma	ICP-MS	897	-	Cu levels in cases (838 μg/L) were significantly lower than in health controls (897 μg/L)
[104]	Case–control	Iran	50 pregnant women with GDM and 50 healthy pregnant women as a control group	Serum from arterial blood	NP	1666 ± 442	-	No significant difference in serum Cu levels between cases (1671 ± 336 μg/L) and controls (1666 ± 442 μg/L).
[105]	Case–control	Iran	46 pregnant women with impaired glucose tolerance and 35 healthy pregnant women	Serum	Spectrophotometry	805 ± 386	-	Cu levels in pregnant women with impaired glucose tolerance (476 ± 204 μg/L) were significantly lower than in controls (804 ± 386 μg/L).
[106]	Case–control	China	251 women divided in four groups: 1. Normal pregnant women 2. Group pregnant women with impaired glucose tolerance 3. Group Pregnant women with GDM, and 4. Group: normal non pregnant women	Serum	ICP-AES	1700		Cu levels were significantly higher in pregnant women (1700 ± 260 μg/L) than in non-pregnant women (914 ± 86 μg/L). No significant difference was found between women with impaired glucose tolerance (1790 ± 360 μg/L) and normoglycemic pregnant women. Women with GDM showed significantly higher Cu levels (1960 ± 560 μg/L) compared to controls (1700 ± 260 μg/L).
[107]	Case–control	China	192 cases with PTB and 282 women with full term delivery. Measuring was performed in 1st and 2nd trimester	Serum	ICP-MS	1824		Copper levels in cases (1795 μg/L) were not significantly different from control group (1824 μg/L).
[108]	Case–control	Malawi	Cases were defined as 91 preterm pregnancies of gestation 26–37 weeks while control were 90 women with full term	Serum	ICP-MS	2390	-	Copper levels in women who had PTB (2610 μg/L) were significantly higher than in women who delivered at term (2390 μg/L).

Abbreviations: *N*—total number of participants; ICP-MS—inductively coupled plasma mass spectrometry; PPROM—preterm premature rupture of membranes; GDM—Gestational diabetes; PTB—Preterm birth; PE—preeclampsia; NTD—Neural tube defects; CHD—congenital heart defects.

**Table 4:** Zinc (Zn) levels in maternal blood and cord blood of pregnant women worldwide (µg/L).

Reference	Study Design	Country	*N*	Biological Material	Analytical Technique	Zn Levels (µg/L)		Main Findings
						Maternal	Cord	
[42]	Cross-sectional	Cairo, Egypt	150 (40 preterm + 35 full-term) and their mothers	Serum	FAAS	Preterm: 960 ± 150 Full-term: 1090 ± 150	Preterm: 730 ± 130 Full-term: 880 ± 180	Maternal and cord serum Zn differed significantly and were positively correlated (ρ = 0.644). Cord Zn did not differ between AGA and SGA infants but was positively associated with gestational age (ρ = 0.305), birth weight, length, and head circumference. Maternal serum Zn was also positively correlated with gestational age and neonatal anthropometric measures, with higher maternal Zn linked to better outcomes
[36]	Cross-sectional	Kuwait	39	Blood	GFAAS	696 ± 34.3	1112 ± 60.9	Cord Zn was higher than maternal blood (maternal/cord ratio: 1.55 ± 0.08). Birth weight was not associated with Zn, but cord Zn was negatively linked to placental weight (450–800 g). The study suggests transplacental Zn transfer occurs via active transport and that blood Zn is not a reliable marker of fetal growth
[52]	Cross-sectional	Jordan	186	Serum	AAS	680 ± 100	1140 ± 230	Serum Zn was lower in cord blood than maternal blood and declined from the 1st to 3rd trimester. Cord Zn was positively correlated with birth weight (ρ = 0.723). In the 3rd trimester, anemic women (Hb < 11 g/dL) had lower serum Zn than non-anemic controls, and cord Zn remained positively associated with birth weight in both groups
[77]	Cross-sectional	Valencia, Spain	54	Plasma	ICP-MS	757 ± 150	692 ± 153	No significant association between cord blood Zn levels with SGA (n = 11), AGA (n = 30), or LGA infants (n = 13).
[33]	Cross-sectional	Tarragona, Spain	53	Blood	SF-ICP-MS	6708	2311	Zn levels were significantly lower in cord than in maternal blood, indicating limited. Zn levels were significantly higher at delivery compared to 1st trimester (6708 μg/L vs. 6147 μg/L). No significant correlation between maternal blood and cord blood Zn levels.
[53]	Cross-sectional	Arctic Canada	352	Plasma	F-AAS	555	986	Cord blood Zn levels were significantly higher than in maternal blood.
[78]	Cross-sectional	Canary Islands, Spain	471	Blood	ICP-MS	-	1179 ± 417	No significant association between cord Zn levels and birth weight.
[47]	Prospective	Bangladesh	745	Serum	ICP-MS	-	1209	No significant association between cord blood Zn levels and birth weight.
[54]	Cross-sectional	Spain and Belgium	66	Serum	F-AAS	1548 ± 848	1959 ± 627	Cord Zn levels were higher than maternal levels, with a significant negative correlation (r = −0.35). The maternal Cu/Zn ratio was higher than in cord blood and positively correlated with maternal age. Maternal Zn increased with gestational age, but Zn was not associated with birth weight
[79]	Case–control	Sudan	104	Serum	F-AAS	Vaginal delivery: 870 Cesarean delivery: 761	Vaginal delivery: 978 Cesarean delivery: 815	Maternal and cord serum Zn were higher after vaginal delivery than cesarean section. Cord Zn was significantly associated with maternal levels, though no overall correlation between maternal and cord serum Zn was observed
[80]	Cross-sectional	France	510 (262 full-term + 248 preterm)	Serum	FAAS	-	Full-term: 1234 ± 202 Preterm: 1332 ± 248	Cord blood Zn was higher in preterm than term infants, with no effect of gender. Zn levels decreased from 26 to 42 weeks of gestation, showing a negative biphasic pattern significantly associated with 26–34 weeks
[81]	Cross-sectional	Benin	60	Blood	ICP-MS	5415	2276	Zinc levels were significantly lower in cord than in maternal blood.
[48]	Prospective	Bangladesh	745	Serum	ICP-MS	-	1209	Zinc levels in cord blood were not associated with the risk of preterm birth.
[28]	Cross-sectional	Jakarta, Indonesia	51	Serum	ICP-MS	Term: 452 Preterm: 403	Term: 2938 Preterm: 3214	No differences in cord blood Zn levels between the preterm group (n = 26) and the group born at term (n = 25).
[83]	Case–control	NP	43 cases with severe PE and 80 healthy pregnant women	Serum	ICP-MS	Cases: 685 ± 875 Cases: 534 ± 139	Cases: 951 ± 230 Controls: 492 ± 135	Zn levels were significantly higher in cord blood of the PE group than in the control group. Zn levels were not significantly different in maternal blood of the two groups.
[69]	Cross-sectional	Zabol, Iran	60	Serum	AAS	684 ± 99	841 ± 110	Serum Zn levels decreased significantly during pregnancy (1st to 3rd trimester) (749 ± 91 vs. 731 ± 106 vs. 684 ± 99 µg/L). Serum Zn levels in cord blood from normal birth weight infants were significantly higher than in cord blood from low birth weight infants.
[45]	Cross-sectional	Bangladesh	44	Plasma	ICP-MS	-	3000	Cord blood Zn levels were significantly and positively associated with birth weight (ρ = 0.35), chest circumference (ρ = 0.36), gestational age (ρ = 0.37), birth length (ρ = 0.39), and head circumference (ρ = 0.32).
[84]	Cross-sectional	Greater Poland region	64	Serum	F-AAS	630 ± 170	650 ± 160	Maternal serum Zn levels decreased significantly after delivery (460 ± 160 µg/L) compared with the period immediately before delivery (630 ± 170 µg/L). Cord serum Zn levels were significantly higher than maternal serum after delivery.
[85]	Cross-sectional	Gryfino, Poland	136	Blood	ICP-OES	2940 ± 1100	1370 ± 570	Maternal Zn levels were over 2-fold higher than those in cord blood (*p* < 0.05) and higher in women who smoked (4230 vs. 2950 μg/L). Cord Zn was positively associated with gestational age and negatively correlated with head circumference
[72]	Prospective cohort	NP	68	Serum	FEAS	1360 ± 740	1570 ± 450	Zn levels were lower in maternal (800 ± 300 μg/L) and cord serum (170 ± 0.430 μg/L) in premature infants with PPROM compared to those without PPROM (1360 ± 740 μg/L vs. 1570 ± 450 μg/L).
[51]	Prospective case–control	Israel	80	Plasma	ICP-MS	Active: 534 ± 139 Elective CD: 617 ± 266	Active: 492 ± 135 Elective CD: 353 ± 120	Significantly higher levels of Zn in cord blood during active labor compared to elective cesarean delivery (CD).
[86]	Cross-sectional	Ma’anshan, China	3416	Serum	ICP-MS	the 1st trimester: 1020 the 2nd trimester: 810	900	No significant difference in Zn levels between maternal and cord blood. A significant difference in Zn levels between the two trimesters of pregnancy.
[49]	Case–control	Varanasi, India	33 cases and 18 controls	Plasma	Spectrophotometry	-	830 ± 88.5	Zn levels were significantly lower in cord blood from PE (n = 19) (744 ± 150 µg/L) and eclampsia (n = 14) (677 ± 117 µg/L) than in the control group (830 ± 88.5 µg/L).
[87]	Cross-sectional	Terai, Nepal	100	Blood	ICP-MS	-	2112	Cord blood Zn levels were not associated with maternal age, socioeconomic status, living environment, and tobacco smoking.
[77]	Cross-sectional	Manhasset, New York, USA	35	Plasma	AAS	625 ± 183	870 ± 95	No significant differences in maternal plasma Zn levels depending on gestational age. Cord plasma Zn levels decreased with gestational age (from 24 to 42 weeks).
[35]	Cross-sectional	South Africa	62	Blood	ICP-MS	6290	2548	No correlation between maternal and cord blood Zn levels.
[26]	Prospective cohort	China	1568	Serum	AAS	5521 ± 939	-	Serum Zn remained stable across trimesters and compared to pre-pregnancy. Levels were significantly lower in cases of miscarriage, preterm labor, and premature rupture of membranes compared to controls.
[41]	Cross-sectional	Lucknow, India	54	Blood	FAAS	5670 ± 2490	9460 ± 8350	Women aged 24–28 had higher blood Zn than those aged 18–23. Maternal Zn was higher and cord Zn slightly lower in low birth weight infants, but not significantly. Maternal and cord Zn did not differ by gestational age
[107]	Cross-sectional	Zhejiang, China	357	Plasma	ICP-MS	-	955	Cord blood Zn levels were significantly higher in babies born in summer than in babies born in autumn/winter.
[39]	Cross-sectional	Serbia	125	Plasma	ICP-MS	484 ± 145	356 ± 138	A marginally significant difference in Zn levels between maternal blood and cord blood. A positive correlation between Zn levels in paired maternal and cord blood samples.
[90]	Longitudinal	Khoy, Iran	162	Serum	ICP-MS	653 ± 149	854 ± 166	Differences in Zn levels during the 1st, 2nd, and 3rd trimesters, 795 ± 150, 745 ± 161, and 653 ± 149 µg/L, respectively. About 42% of pregnant women were Zn deficient. Zn levels in cord serum were significantly higher than in maternal serum.
[21]	Case–control	Harran, Turkey	144	Serum	AAS	Cases: 836 ± 334 Controls: 1036 ± 300	Cases: 1391 ± 504 Controls: 1294 ± 346	Maternal serum Zn levels in the NTD group were significantly lower than in the control group.
[91]	Cross-sectional	Seville, Spain	100	Blood	ICP-MS	5088	2221	Zm levels in maternal blood were nearly three times higher than in cord blood (*p* < 0.05). Zn levels were positively correlated between maternal and cord blood.
[92]	Cross-sectional	Lublin, Poland	134	Blood	HR-ICP-OES	-	2025 ± 718	No significant correlations in Zn levels with anthropometric parameters.
[91]	Prospective cohort	Peking, China	48	Plasma	ICP-MS	597	687	No significant differences in plasma Zn levels during pregnancy (from the 1st to the 3rd trimester). Significantly higher Zn levels in cord plasma than in maternal plasma.
[93]	Prospective cohort	Norway	211	Blood	SF-ICP-MS	5110	-	Blood Zn levels increased significantly during pregnancy, 3 days postpartum, and 6 weeks postpartum.
[109]	Cross-sectional	Taipei, Taiwan	308	Serum	ICP-MS	-	6192 ± 2508	Maternal blood Zn levels reduce transplacental Pb transfer; a 10% increase in maternal blood Zn levels contributes to a 0.37 µg/L decrease in cord Pb levels.
[95]	Longitudinal	Shanghai, China	100	Blood (at 4 time points: preconception, GW 16, 24, and 32)	ICP-MS	6060 ± 910	1940 ± 480	Maternal blood Zn was higher before conception (5470 μg/L) than at 16 weeks (5070 μg/L) and remained relatively stable thereafter. Preconception Zn levels were correlated with levels at 24 weeks and with cord blood Zn
[43]	Case–control	Nottingham, England	55 cases with PE, 60 healthy normotensive pregnant women, and 30 healthy non-pregnant women	Plasma	ICP-MS	486	772	Non-pregnant women (635 µg/L) had higher plasma Zn than normotensive pregnant women. Maternal Zn was lower in PE (418 µg/L) than controls (486 µg/L), with both early- and late-onset PE showing significant reductions. Cord plasma Zn was higher than maternal levels in both groups but did not differ between PE (830 µg/L) and controls (772 µg/L), nor by PE onset. Maternal Zn was positively correlated with birth weight.
[31]	Cross-sectional	Enugu Metropolis, Nigeria	48	Serum	AAS	416 ± 24.5	427 ± 47.0	Compared to the Zn reference ranges they found in the literature (660–1100 µg/L for maternal blood and >65 µg/dL for cord blood), the authors concluded that their study population had a Zn deficiency.
[30]	Case–control	Kermanshah province, Iran	57 cases with diabetes and 54 controls	Serum	ICP-MS	Cases: 397 ± 531 Controls: 334 ± 694	-	No significant differences in maternal Zn blood levels between cases and controls.
[59]	Case–control	Guangdong, China	515 cases with PTB and 595 controls	Blood	ICP-MS	-	Cases: 1390 Controls: 1580	Cord blood Zn levels were significantly lower in cases than in controls. Cord blood Zn levels were negatively associated with PTB.
[34]	Cross-sectional	Argentina	696	Blood	ICP-MS	6682	-	Blood Zn levels and parity were positively associated. Negative associations between blood Zn levels and birth weight, birth length, and head circumference. Maternal blood Zn levels were positively associated with gestational age in female children.
[22]	Cross-sectional	Isfahan, Iran	263	Blood (collected during the 1st trimester)	ICP-MS	2518 ± 1484	-	Blood Zn levels were positively associated with parity and, in female infants, with gestational age. Negative associations were observed with birth weight, length, and head circumference, though overall, Zn was not significantly linked to birth size outcomes
[95]	Retrospective	Guangzhou, China	11,222 pregnant women and 545 non-pregnant women	Blood	ICP-MS	5700	-	Compared to non-pregnant women, Zn levels decreased during pregnancy.
[98]	Observational case–control	Istanbul, Turkey	88 pregnant women (in their 3rd trimester) included 43 PE patients and 45 normotensive pregnant women as controls.	Serum	Spectrophotometry	803	-	No significant difference in Zn levels between PE women (751 ± 209 μg/L) and controls (803 ± 177 μg/L).
[29]	Case–control	Sudan	50 PE and 50 healthy pregnant women	Serum	AAS	1080	-	No significant difference in Zn levels between PE women (1080 μg/L) and controls (1020 μg/L).
[100]	Case–control	Sanliurfa, Turkey	24 women with PE and 44 women with normotensive pregnancies	Plasma	AAS	11.93 ± 3.11 µg/g protein	-	Significantly higher Zn levels in PE group (15.53 ± 4.92 µg/g protein) than in controls (11.9 ± 3.11 µg/g protein).
[24]	Case–control	Nnewi, Nigeria	54 cases with PE, 48 non-PE pregnant controls	Serum	AAS	540 ± 39	-	Levels of serum Zn in PE women (801 ± 119 µg/L) were significantly higher than in controls (540 ± 39 µg/L).
[100]	Case–control	Gorgan, Iran	50 cases with PE and 50 healthy pregnant controls	Serum	AAS	730 ± 330	-	Zinc levels in PE women (710 ± 260 μg/L) were not significantly different than in controls (730 ± 330 μg/L).
[19]	Case–control	Bangladesh	50 cases with PE and 58 normotensive pregnant women	Serum	AAS	980 ± 30.0	-	Levels of serum Zn in PE women (770 ± 50 μg/L) were significantly lower than in controls (980 ± 30 μg/L).
[110]	prospective cohort	Poznan, Poland	121 with pregnancy-induced hypertension (10–14 gestational weeks) and 363 normotensive pregnant controls	Serum	ICP-MS	615	-	Levels of serum Cu in pregnant women with PIH (615 μg/L) were not significantly different from controls (615 μg/L). Serum Zn levels in the 10th–14th week of pregnancy were not associated with pregnancy-induced hypertension.
[27]	Cross-sectional	Guangdong Province, China	97 with mild PE, 64 with severe PE, and 110 were normotensive healthy pregnant controls	Serum	ICP-MS	5795		Zn levels were lowest in the severe PE group (3956 μg/L), significantly below both the control (5795 μg/L) and mild PE (7502 μg/L) groups, while the mild PE group had significantly higher Zn than controls
[96]	Cross sectional	Dhaka, Bangladesh	44 PE women, 33 with eclampsia, and 27 normotensive pregnant women	Serum	AAS	980 ± 130	-	Serum Zn levels were significantly higher in PE (1044 ± 130 µg/L) than in eclampsia (914 ± 130 µg/L). Serum Zn levels in controls (980 ± 130 µg/L) did not differ compared to other groups.
[102]	Case–control	Ankara, Turkey	14 pregnant women with babies with NTDs in 2nd trimester and 14 controls	Serum	AAS	1026 ± 237 µg/L	-	Serum Zn levels were significantly lower in cases (624.8 ± 159 µg/L) than in controls (1026 ± 237 µg/L).
[111]	Case–control	Iran	23 women who gave birth to babies with NTD and 36 controls with healthy neonates	Serum	AAS	877	-	Serum Zn levels in women with NTD neonates (740 µg/L) were significantly lower than in the controls (870 µg/L).
[112]	Case–control	Dhaka, Bangladesh	32 women who gave birth to babies with NTD and 32 controls with healthy neonates	Serum	AAS	883 ± 65.0	-	Serum Zn levels in women with NTD babies (610 ± 53.1) µg/L were significantly lower than in controls (883 ± 65.0 µg/L).
[104]	Case–control	Iran	50 pregnant women with GDM and 50 controls	Serum from arterial blood	NP	676	-	No significant difference between Zn levels in serum of pregnant women with GDM (692 μg/L) and controls (676 μg/L).
[105]	Case–control	Iran	46 pregnant women with impaired glucose tolerance and 35 healthy pregnant women	Serum	Photometric technique	720 ± 301	-	Serum Zn levels in pregnant women with impaired glucose tolerance (610 ± 204 µg/L) were significantly lower than in controls (720 ± 301 µg/L).
[113]	Case–control	Dhaka Bangladesh	80 pregnant women with GDM and 80 healthy pregnant women	Serum	Spectrophotometry	918 ± 382	-	Maternal serum Zn levels in the group with GDM (596 ± 19.3 µg/L) were significantly lower than controls (918 ± 38.1 µg/L).
[106]	Case–control	China	251 women divided into four groups: 1. Normal pregnant women (NPW); 2. Pregnant women with impaired glucose tolerance (IGT); 3. Pregnant women with GDM; and 4. Normal non pregnant women (NW)	Serum	ICP-AES	1130	-	Serum Zn levels in women with GDM (1020 μg/L) were lower than in women with normal pregnancy (1130 μg/L). Serum Zn was highest in the NW group (1430 ± 300 μg/L), significantly above the NPW group (1130 ± 330 μg/L). The IGT (1080 ± 270 μg/L) and GDM (920 ± 190 μg/L) groups had lower Zn than NPW, but the differences were not statistically significant.
[107]	Case–control	China	192 cases with PTB and 282 women with full term delivery. Measurements in 1st and 2nd trimesters	Serum	ICP-MS	813	-	No significant difference in Zn levels between cases (828 μg/L) and controls (813 μg/L).
[108]	Case–control	Malawi	91 cases who spontaneously delivered preterm babies with gestation 26–37 weeks and 90 healthy pregnant women with term deliveries	Serum	ICP-MS	730	-	Serum Zn levels were significantly higher in cases (810 μg/L) than in controls (730 μg/L)

Abbreviations: *N*—total number of participants; ICP-MS—inductively coupled plasma mass spectrometry; GDM—Gestational Diabetes melitus; PE—preeclampsia; PTB—Preterm birth; NPW—normal pregnant women; NTD—Neural tube defects; IGT—impaired glucose tolerance; FEAS—flame atomic emission spectrophotometer.

**Table 5:** Selenium (Se) levels in maternal blood and cord blood of pregnant women worldwide (µg/L).

Reference	Study Design	Country	*N* Total, or Cases and Controls	Biological Material	Analytical Technique	Se Levels (µg/L)		Main Findings
						Maternal	Cord	
[36]	Cross-sectional	Kuwait	39	Blood	GFAAS	107 ± 6.90	80.4 ± 2.60	Maternal Se levels were significantly higher than cord blood (maternal/cord ratio: 0.83 ± 0.04 μg/L). Birth weight was not associated with Se levels. Results suggest that transplacental Se transfer occurs via passive diffusion
[77]	Cross-sectional	Valencia, Spain	54	Plasma	ICP-MS	56 ± 15.7	34.6 ± 6.21	No significant association between cord blood Se levels with SGA (n = 11), AGA (n = 30), or LGA infants (n = 13).
[33]	Cross-sectional	Tarragona, Spain	53	Blood	SF-ICP-MS	107	100	Maternal blood Se levels were significantly lower at delivery compared to the 1st trimester (107 vs. 114 μg/L). Se levels in maternal and cord blood were not significantly correlated.
[53]	Cross-sectional	Arctic Canada	352	Plasma	GF-AAS	120	78	Maternal blood Se levels were significantly higher than in cord blood.
[78]	Cross-sectional	Canary Islands, Spain	471	Blood	ICP-MS	-	66.7 ± 24.3	No significant relationship between cord Se levels and birth weight.
[47]	Prospective	Bangladesh	745	Serum	ICP-MS	-	15.8	Cord blood Se levels were positively associated with birth weight.
[23]	Case–control	Sweden	80	Erythrocytes	ICP-MS	102	78.2	No significant differences in Se levels between maternal and cord blood. Se levels in maternal and cord erythrocytes did not differ in pregnant women with anthroposophic lifestyle (n = 40) compared to those without (n = 40).
[80]	Cross-sectional	France	248 preterm and 262 full-term neonates	Serum	GFAAS	-	Full-term: 47.4 ± 7.90 Preterm: 39.5 ± 7.90	Cord blood Se levels were significantly lower in preterm than in term neonates. No significant effect of gender on cord blood Se levels. Se had a biphasic pattern with a significant positive correlation between 26 and 38 weeks.
[114]	Cross-sectional	Beijing, Lanzhou, Taiyuan, and Xiamen, China	81	Serum	ICP-MS	141	131	No significant differences in Se levels between maternal and cord blood. No significant differences between Se levels in maternal blood or cord blood and birth weight.
[48]	Prospective	Bangladesh	745	Serum	ICP-MS	-	15.8	Se levels in cord blood were not associated with the risk of preterm birth.
[115]	Cross-sectional	Tokyo, Japan	24	Blood	ICP-MS	-	6.70	No significant difference in the levels of Se in cord blood and the hormones TSH and free T4.
[28]	Cross-sectional	Jakarta, Indonesia	26 preterm and 25 term pregnancies	serum	ICP-MS	Term: 76.4 Preterm: 72.8	Term: 49.7 Preterm: 41.8	Se levels were significantly higher in cord serum from the term group than from the preterm group.
[83]	Case–control	NP	43 cases with severe PEand 80 healthy pregnant women	Serum	ICP-MS	Cases: 99.9 ± 24.9 Controls: 115 ± 17.3	Cases: 82.8 ± 17.2 Controls: 105 ± 24.9	Selenium levels were significantly lower in maternal and cord blood in the PE group than in the control group.
[45]	Cross-sectional	Bangladesh	44	Plasma	ICP-MS	-	190	Cord blood Se levels were not significantly associated with birth weight, chest circumference, gestational age, birth length, or head circumference.
[51]	Prospective case–control	Israel	80	Plasma	ICP-MS	Active: 115 ± 17.3 Elective CD: 106 ± 20.6	Active: 105.0 ± 24.9 Elective CD: 109 ± 36.7	Significantly higher levels of Se in maternal plasma during active labor compared to elective cesarean delivery (CD).
[85]	Cross-sectional	Ma’anshan, China	3416	Serum	ICP-MS	the 1st trimester: 72.8 the 2nd trimester: 70.3	43.2	Se levels in cord serum were significantly lower than in maternal serum. A significant difference in Se levels between the two trimesters of pregnancy.
[116]	Cross-sectional	Haifa, Israel	168	Serum	AAS	86.8 ± 27.5	68.4 ± 26.6	Cord serum Se levels increased with gestational age, particularly after 36 weeks, and were significantly associated with birth weight and 5 min Apgar score, but not with maternal characteristics or complications. Multiple regression confirmed gestational age as a significant predictor of cord Se. In contrast, maternal serum Se levels did not vary with gestational age, study groups, or maternal and neonatal outcomes
[49]	Case–control	Varanasi, India	33 PE cases and 18 controls	Plasma	Spectrophotometry	-	22.2 ± 4.19	Se levels were significantly lower in cord blood in PE (n = 19) (18.6 ± 5.21 μg/L) and eclampsia (n = 14) (16.3 ± 5.23 μg/L) than in controls (22.2 ± 4.19).
[87]	Cross-sectional	Terai, Nepal	100	Blood	ICP-MS	-	169	Cord blood Se levels were not significantly associated with maternal age, socioeconomic status, living environment, and tobacco smoking.
[35]	Cross-sectional	South Africa	62	Blood	ICP-MS	104	111	A significant correlation between Se levels in maternal and cord blood.
[117]	Cross-sectional	Kumamoto, Japan	54	Blood	ICP-MS	156 ± 21.7	176 ± 22.9	Se levels in cord blood were slightly but significantly higher than in maternal blood. Selevels in cord blood and maternal blood were significantly and positively correlated.
[39]	Cross-sectional	Serbia	125	Plasma	ICP-MS	135 ± 30.9	72.0 ± 25.1	A significant difference in Se levels between maternal and cord blood. No positive correlation between paired maternal and cord blood samples.
[55]	Cross-sectional	Jiangsu province, eastern China	209	Blood	ICP-MS	131	126	Maternal Se levels were higher than cord blood and significantly correlated (ρ = 0.29). Se was positively associated with birth weight and negatively correlated with cord Cd, suggesting a potential protective effect.
[118]	Cross-sectional	Shengsi Islands, China	106	Serum	ICP-MS	-	384 ± 338	Se levels were significantly and positively correlated between cord blood and colostrum (ρ = 0.40). Se levels were marginally and negatively associated with reduced head circumference at birth.
[21]	Case–control	Harran, Turkey	144	Serum	AAS	Cases: 46.8 ± 26.4 Controls: 47.6 ± 20.6	Cases: 42.2 ± 21.9 Controls: 39.9 ± 20.0	No significant differences in maternal and cord blood Se levels between NTD cases and controls.
[90]	Cross-sectional	Seville, Spain	100	Blood	ICP-MS	120	118	Se levels did not differ significantly between maternal and cord blood. Se levels in maternal and cord blood were positively correlated.
[92]	Cross-sectional	Lublin, Poland	134	Blood	HR-ICP-OES	-	158 ± 41.6	No significant differences in Se levels with anthropometric parameters.
[91]	Prospective cohort	Peking, China	48	Plasma	ICP-MS	95.3	53.6	Significant decreases in maternal plasma Se levels from the 1st, 2nd to 3rd trimesters (111 vs. 103 vs. 95.3 µg/L). Significantly lower Se levels in cord plasma than in maternal plasma.
[93]	Prospective cohort	Norway	211	Blood	SF-ICP-MS	85.0	-	No significant differences in blood Se levels between the 2nd trimester, 3 days postpartum, and 6 weeks postpartum. Blood Se levels were lowest 3 days postpartum. Fish consumption, especially shellfish, was a strong positive predictor of elevated maternal blood Se levels.
[94]	Longitudinal	Shanghai, China	100	Blood (at 4 time points: preconception, GW 16, 24, and 32)	ICP-MS	140 ± 28.8	115 ± 25.1	Maternal blood Se was higher before conception (137 μg/L) than at 16 weeks (131 μg/L) and remained relatively stable thereafter. Se levels were correlated between preconception and 24 weeks, and maternal preconception Se was positively associated with cord blood Se throughout pregnancy and at birth.
[43]	Case–control	Nottingham, England	55 cases with PE, 60 healthy normotensive pregnant women, and 30 healthy non-pregnant women	Plasma	ICP-MS	59.5	42.7	Maternal plasma Se was significantly lower in women with PE (51.4 µg/L) than in normotensive controls (59.5 µg/L), while non-pregnant women had higher levels (median 78.9 µg/L). Cord plasma Se was lower than maternal Se and further reduced in newborns of PE cases (37.4 µg/L) versus controls (42.7 µg/L). These differences persisted for both early- and late-onset PE. Maternal Se levels were also positively correlated with birth weight.
[31]	Cross-sectional	Enugu Metropolis, Nigeria	48	Serum	AAS	168 ± 14.5	198 ± 16.7	Compared to the Se reference ranges they found in the literature (70–250 µg/L for maternal blood and 35–107 µg/L for cord blood), the authors concluded that their study population had a Se overload.
[30]	Case–control	Kermanshah province, Iran	57 cases with diabetes and 54 controls	Serum	ICP-MS	Cases: 75.2 ± 47.4 Controls: 60.6 ± 44.0	-	No significant differences in maternal blood Se levels in cases and controls.
[59]	Case–control	Guangdong, China	515 cases with preterm births (PTB) and 595 controls	Blood	ICP-MS	-	Cases: 93.6 Controls: 97.7	Cord blood Se levels were significantly lower in cases than controls. Cord blood Se levels were negatively associated with PTB. The risk of PTB decreased, followed by a leveling off with increasing Se levels.
[34]	Cross-sectional	Argentina	696	Blood (collected 36 ± 12 h postpartum)	ICP-MS	129	-	Se was positively associated with parity and linked to birth outcomes, showing negative associations with neonatal anthropometric measurements
[68]	Case–control	South-East Nigeria.	116 cases with PE and normotensive pregnant controls	Serum	AAS	94.8 ± 36.34	-	Serum Se levels in controls (94.8 ± 36.3 µg/L) were significantly higher than in PE group (52.9 ± 21.3 µg/L).
[116]	Case–control	Shiraz, Iran	38 cases with preeclampsia (PE) and 612 controls (pregnant primigravida women)	Serum	AAS	70.6 ± 21.4	-	Maternal serum Se levels in PE group (70.63 ± 21.41 µg/L) were lower than controls (82.0 ± 15.5 µg/L).
[40]	Case–control	Bangladesh	74 cases with preeclampsia (52 mild and 22 severe) and 118 normotensive pregnant women	Serum	AAS	32.2 ± 1.22	-	Se levels in cases (23.8 ± 0.64 µg/L) were significantly lower than controls (32.2 ± 1.22 µg/L).
[110]	Prospective cohort	Poznan, Poland	121 women with pregnancy-induced hypertension (PIH) and 363 normotensive women (10–14 week of pregnancy)	Serum	ICP-MS	62.9	-	Serum Se was 62.9 µg/L in cases and 57.5 µg/L in women who developed PIH. Lower Se levels in early healthy pregnancy were associated with higher PIH risk and had strong prognostic value.
[9]	Case–control	Tehran, Iran	91 women who delivered LBW neonates and 86 women who delivered normal birth weight neonates	Blood	NP	78.5 ± 25.5		Maternal Se levels (cases: 80.7 ± 28.0 μg/L; controls: 78.5 ± 25.5 μg/L) and cord blood Se levels (cases: 77.3 ± 26.1 μg/L; controls: 73.9 ± 24.4 μg/L) did not differ significantly between groups. A significant correlation was observed between maternal and cord Se in both cases and controls.
[102]	Case–control	Ankara, Turkey	14 pregnant women with NTDs fetuses in the 2nd trimester, and 14 pregnant women with normal babies as a control group	Serum	AAS	77.4 ± 5.50	-	Serum Se levels were significantly lower in cases (55.2 ± 11.3 µg/L) than controls (77.4 ± 5.50 µg/L).
[37]	Case–control	Tanzania	287 women with neonates with NTDs and a randomly selected control group	Blood Serum	Spectrophotometer	Blood Se: 364 ± 17.1 Serum Se: 77.4 ± 5.50	-	Serum Se was significantly lower in cases (55.2 ± 11.3 μg/L) than in controls (77.4 ± 5.5 μg/L), and whole-blood Se was also significantly lower in cases (306 ± 10.95 μg/L) compared to controls (364 ± 17.1 μg/L).
[103]	Case–control	China	112 pregnant women with fetuses with CHD and 109 healthy pregnant women as controls	Blood	ICP-MS	186 L	-	Se levels in blood of cases (172 μg/L) were significantly lower than in controls (186 μg/L).
[104]	Case–control	Iran	50 pregnant women with GDM and 50 as controls	Serum from arterial blood	NP	94.7 ± 17.8	-	No significant difference between Se serum levels in cases (98.2 ± 22.8 μg/L) and controls (94.7 ± 17.8 μg/L).
[107]	Case–control	China	192 cases with PTB and 282 women with full term delivery. Measurements were taken in 1st and 2nd trimesters	Serum	ICP-MS	96.8	-	No significant difference between Se serum levels in cases (99.8 μg/L) and controls (96.8 μg/L).
[9]	Case–control	Malawi	91 cases with spontaneous preterm deliveries (gestations of 26–37 weeks) and 90 full term deliveries	Serum	ICP-MS	74.2	-	Serum Se tended to be higher in spontaneous preterm births (79.7 µg/L) than at-term deliveries (74.2 µg/L), without statistical significance.
[119]	Case–control	China	98 women with IGT; 46 with GDM; and 90 normal pregnant women (NPW).	Serum	AAS	74.1 ± 17 µg/L		Serum Se was significantly lower in pregnant women with GDM than in healthy controls (74.1 ± 17 µg/L)

Abbreviations: *N*—total number of participants; ICP-MS—inductively coupled plasma mass spectrometry; GDM—gestational diabetes mellitus; IGT—Impaired glucose tolerance; PE—preeclampsia; PTB—Preterm birth; NPW—normal pregnant women; NTD—Neural tube defects; CHD—congenital heart defects.

## Data Availability

No new data were created or analyzed in this study. Data sharing is not applicable to this article.
